# CDC20‐Mediated Selective Autophagy Degradation of PBRM1 Affects Immunotherapy for Renal Cell Carcinoma

**DOI:** 10.1002/advs.202412967

**Published:** 2024-12-10

**Authors:** Yizeng Fan, Weichao Dan, Taotao Que, Yi Wei, Bo Liu, Zixi Wang, Yulin Zhang, Yuzhao Wang, Tianjie Liu, Yanxin Zhuang, Mengxing Li, Chendong Guo, Jin Zeng, Bohan Ma, Lei Li

**Affiliations:** ^1^ Department of Urology The First Affiliated Hospital of Xi'an Jiaotong University Xi'an Shaanxi 710061 P. R. China; ^2^ Key Laboratory of Environment and Genes Related to Diseases Ministry of Education Xi'an Shaanxi 710061 P. R. China; ^3^ Key Laboratory for Tumor Precision Medicine of Shaanxi Province The First Affiliated Hospital of Xi'an Jiaotong University Xi'an Shaanxi 710061 P. R. China

**Keywords:** CDC20, Immunotherapy, p62, PBRM1, RCC

## Abstract

Polybromo 1 (PBRM1) inactivating mutations are associated with clinical benefit from immune checkpoint inhibitor treatments in clear cell renal cell carcinoma (ccRCC). However, whether targeting PBRM1 has the potential to enhance immunotherapy efficacy in patients with wild‐type PBRM1 and the upstream pathways that regulate PBRM1 protein stability remain unclear. Here, it is demonstrated that PBRM1 knockdown induced M1 macrophage polarization and infiltration, which enhanced the efficacy of anti‐PD‐1 immunotherapy in RCC. Meanwhile, CDC20 catalyzes K27 ubiquitination of PBRM1 and promotes its degradation via p62‐mediated selective autophagy. A bicyclic peptide (PB1‐p62) is designed and constructed to target PBRM1 and p62, thereby promoting the degradation of PBRM1. As a result, the efficacy of anti‐PD‐1 immunotherapy is enhanced, leading to improved overall survival rates in syngeneic mouse tumor models. Overall, this finding suggest the clinical application of PB1‐p62 and provide a novel approach for enhancing the effectiveness of immunotherapy in RCC patients with wild‐type PBRM1.

## Graphical abstract

This study found that Polybromo‐1 (PBRM1) sensitizes renal cell carcinoma (RCC) to immunotherapy through regulating macrophage‐associated chemokines and increasing M1 macrophage infiltration. Furthermore, the APC^CDC20^ E3 ligase is identified as the upstream regulator responsible for p62‐dependent selective autophagy degradation of PBRM1. The inhibition of PBRM1 through a synthesized peptide resulted in increased sensitivity of cancer cells to immunotherapy.

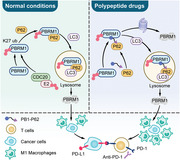

## 1 Introduction

Renal cell cancer (RCC), as one of the most common malignant tumors, has a high incidence and fatality rate worldwide, and it is a major malignant disease that threatens human health.^[^ ^1^ ^]^ Accumulating evidence indicates that the gene mutations are responsible for RCC initiation and progression. Polybromo 1 (PBRM1, also known as PB1 or BAF180), which has 9 major spliceosome types, is a subunit of the SWI/SNF transcription‐modulating chromatin remodeling complex that controls DNA accessibility for transcription. *PBRM1* has been found to be mutated in up to 41% of clear cell renal cell carcinoma (ccRCC), making it the second most commonly mutated gene after *VHL* in ccRCC.^[^ ^2^ ^]^ Additionally, PBRM1 mutation is also observed in several other malignancies, including chordomas (11%–59%), cholangiocarcinomas (12%–23%), and non‐small cell lung cancers (≈3%). In general, PBRM1 was considered as a tumor suppressor, which regulates divergent processes including cell cycle and DNA damage.^[^ ^3^ ^]^ Growing evidence suggests a close association between PBRM1 and immunity. Recently, immunotherapy has been considered as a promising approach for advanced ccRCC, the patients in this stage were too late to surgery and had poor prognosis.^[^ ^4^ ^]^ Previous clinical research has demonstrated that the loss of PBRM1 increases the sensitivity of renal cancer to T cell‐mediated cytotoxic killing.^[^ ^5^ ^]^ PBRM1 status has been significantly associated with the response to anti‐PD‐1 therapy, with a higher proportion of patients with PBRM1 mutations showing a significant benefit in both progression‐free survival (PFS) and overall survival (OS) than patients with PBRM1 wild‐type.^[^ ^6^ ^]^ What we are interested, however, is to enable wild‐type PBRM1 patients to benefit from immunotherapy. Thus, the upper regulatory mechanism of PBRM1 stability is critical for improving the efficiency of immunotherapy in PBRM1 wild‐type patients.

Cell division cycle 20 homologue (CDC20), a well‐known regulator of the cell cycle, controls the correct segregation of chromosomes during mitosis.^[^ ^7^ ^]^ CDC20 and CDC20‐like protein 1 (CDH1) are co‐activators of the anaphase‐promoting complex/cyclosome (APC/C), a multifunctional E3 ubiquitin ligase that participates in the cell cycle, metabolism, DNA damage and repair, autophagy, apoptosis, senescence, and tumorigenesis.^[^ ^8^ ^]^ APC/C and CDC20 play important roles in the transition from metaphase to anaphase during mitosis, ensuring proper chromatid segregation.^[^ ^9^ ^]^ In addition, previous studies have shown that CDC20 plays a key role in different cell types and participates in specific biological processes such as cilia disassembly,^[^ ^10^ ^]^ brain development,^[^ ^11^ ^]^ tissue homeostasis in human keratinocytes,^[^ ^12^ ^]^ genomic stability,^[^ ^13^ ^]^ aging,^[^ ^14^ ^]^ and autophagy.^[^ ^15^, ^16^ ^]^ APC/C^CDC20^ also participates in cellular processes associated with tumorigenesis by regulating REV1,^[^ ^17^ ^]^ Wnt/β‐catenin,^[^ ^18^ ^]^ SOX2,^[^ ^19^ ^]^ MCL‐1,^[^ ^20^ ^]^ PC‐PLC,^[^ ^21^ ^]^ BCL‐2,^[^ ^22^ ^]^ and BIM.^[^ ^23^ ^]^ Recent research has revealed that the expression level of CDC20 plays a crucial role in regulating the anti‐tumor immune system. In hepatocellular carcinoma (HCC), CDC20 expression showed a significant correlation with immune infiltration of B cells, neutrophils, macrophages, and myeloid dendritic cells.^[^ ^24^, ^25^ ^]^ Moreover, the CDC20 protein has been associated with the tumor mutation burden, immune checkpoint molecules, tumor microenvironment, and immunological infiltration in ccRCC.^[^ ^26^ ^]^ However, APC‐dependent molecular mechanism of CDC20 in controlling RCC tumor immunity remains largely unknown.

Autophagy refers to the degradation of various material components in cells through lysosomes, and it can be divided into 3 categories: macroautophagy, microautophagy, and chaperone‐mediated autophagy (CMA).^[^ ^27^ ^]^ Macroautophagy can also be divided into selective and non‐selective forms. After the degradation substrate is labeled during selective autophagy, it is recognized by selective autophagy receptors, of which the most canonical is p62/SQSTM1, and isolated into the autophagosome, where it is degraded when the autophagosome binds to the lysosome to form the autophagolysosome.^[^ ^28^, ^29^ ^]^ p62 is the most extensively studied receptor in selective autophagy, but the regulatory mechanism of p62 has not yet been elucidated.^[^ ^30^ ^]^ As p62 is the first and most important autophagy receptor of selective autophagy, the binding and degradation of p62 to specific substrates will greatly affect target protein level, which also provides new ideas and research directions for the targeted therapy of some clinical diseases.

In our study, we validated that CDC20 catalyzes PBRM1 K27 ubiquitination and degradation via p62‐mediated selective autophagy, and low‐level of PBRM1 enhances the efficacy of immunotherapy through promoting M1 macrophages and CD8^+^ T cells infiltration. Therefore, a peptide named PB1‐p62 was designed to target PBRM1 and p62, resulting in the degradation of most of PBRM1 via autophagy and notably ensuring the efficacy of immunotherapy for renal cancer. Thus, our study shows a new research direction for improving the immune efficacy of RCC in PBRM1 wild‐type patients and greatly improves the possibility of using targeted peptides in the clinical treatment of PBRM1 wild‐type patients.

## 2 Results

### 2.1 CDC20 was Identified as an Interactor of PBRM1

**Figure 1:**
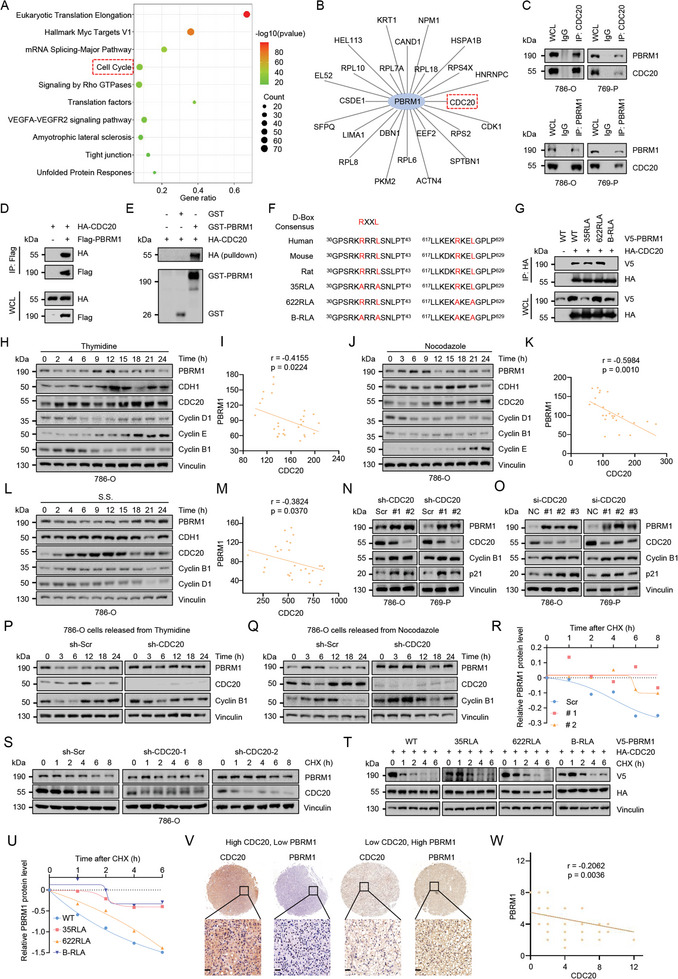
CDC20 directly interacts with and modulates PBRM1. A) anti‐Flag immunoprecipitates derived from 293T cells transfected with Flag‐tagged PBRM1 plasmids coupled with liquid chromatography mass spectrometry (LC‐MS/MS) to identify PBRM1‐interacting proteins in 293T. IPs‐MS results were subjected to enrichment analysis. B) The proteins were enriched in the “cell cycle” categories. C) Immunoblot (IB) analysis of whole cell lysates (WCL) and anti‐CDC20 or anti‐PBRM1 immunoprecipitates derived from 786‐O and 769‐P cells. D) IB analysis of WCL and anti‐Flag immunoprecipitates derived from 293T cells transfected with HA‐CDC20 and Flag‐PBRM1. E) IB analysis of WCL and GST pull‐down products derived from 293T cells transfected with HA‐CDC20 and indicated constructs of GST‐PBRM1. F) Schematic representation of the conserved sites in PBRM1 matching the optimal CDC20 substrate motif. The sequence alignment of from various species as well as a schematic representation of the D‐box mutant using is shown. G) IB analysis of WCL and anti‐Flag immunoprecipitates derived from 293T cells transfected with HA‐CDC20 and indicated constructs of V5‐PBRM1. H) IB analysis of WCL derived from 786‐O cells synchronized by double thymidine block, following by releasing back into the cell cycle for the indicated times. I) Quantification of CDC20 and PBRM1 blot intensity using the ImageJ software, according to Figure 1H. Data are statistics of three independent experiments. CDC20 and PBRM1 bands were normalized to vinculin. J) IB analysis of WCL derived from 786‐O cells synchronized by nocodazole block, following by releasing back into the cell cycle for the indicated times. K) Quantification of CDC20 and PBRM1 blot intensity using the ImageJ software, according to Figure 1J. Data are statistics of three independent experiments. CDC20 and PBRM1 bands were normalized to Vinculin. L) IB analysis of WCL derived from 786‐O cells synchronized by serum starvation (S.S.), following by releasing back into the cell cycle for the indicated times. M) Quantification of CDC20 and PBRM1 blot intensity using the ImageJ software, according to Figure 1L. Data are statistics of three independent experiments. CDC20 and PBRM1 bands were normalized to vinculin. N) Cell lysates derived from 786‐O and 769‐P cells stably expressing shCDC20 or Scr. Scr, Scramble. O) Cell lysates derived from 786‐O and 769‐P cells transfected with siCDC20 or NC. NC, negative control. P) IB analysis of WCL derived from wild‐type (WT) or CDC20 knockdown 786‐O cells synchronized by double thymidine block, following by releasing back into the cell cycle for the indicated times. Scr, Scramble. Q) IB analysis of WCL derived from wild‐type (WT) or CDC20 knockdown 786‐O cells synchronized by nocodazole block, following by releasing back into the cell cycle for the indicated times. R,S) IB analysis and quantification of cell lysates of wild‐type (WT) or CDC20 knockdown 786‐O cells treated with cycloheximide (CHX, 20 µg ml^−1^) at indicated time points. T–U) IB analysis and quantification of cell lysates derived from 293T cells transfected with indicated plasmids and treated with cycloheximide (CHX, 20 µg ml^−1^) at indicated time points. (V–W) Representative images(V) and quantifications(W) of ccRCC patient samples stained for PBRM1 and CDC20 by IHC assay. Scale bar, 20 µm.

To investigate the interactions, we overexpressed PBRM1 by transferring the PBRM1‐Flag plasmid into the 293T cell line and then detected the interacting proteins of PBRM1 via liquid chromatography mass spectrometry (LC‐MS/MS). Enrichment analysis resulted in the discovery of proteins that were enriched in the cell cycle signaling pathway (**Figure** **1**A), with key cell cycle component CDC20 being among the identified cell cycle‐related proteins (Figure 1B). Further co‐immunoprecipitation (Co‐IP) assays confirmed the exogenous and endogenous interactions between PBRM1 and CDC20 (Figure 1C,D; Figure S1A, Supporting Information). Meanwhile, a direct interaction between PBRM1 and CDC20 was demonstrated by a GST pull‐down assay (Figure 1E). As most APC CDC20 substrates contain a destruction box (D‐box) motif RXXL (where R represents Arginine, L represents Leucine, and X represents an arbitrary amino acid), examination of the PBRM1 sequence revealed two putative D‐boxes (Figure 1F). We mutated all the arginine (R) and leucine (L) to alanine (A) and generated three PBRM1 mutant constructs (35RLA, 622RLA and B‐RLA). PBRM1 single mutation (35RLA or 622RLA) did not affect the interaction between PBRM1 and CDC20, whereas simultaneous mutation (B‐RLA) of these sites significantly weakened the interaction between PBRM1 and CDC20 (Figure 1G). Structurally, CDC20 contains a WD40 repeat domain at its C‐terminus that mediates substrate recognition. As shown in Figure S1B,C (Supporting Information), PBRM1 specifically interacted with the WD40‐repeat domain of CDC20, supporting the hypothesis that PBRM1 is a putative CDC20 substrate. In addition, we also found a weak interaction between PBRM1 and CDH1 (Figure S1D, Supporting Information). However, protein purification and GST pull‐down revealed that PBRM1 and CDH1 did not interacted directly (Figure S1E, Supporting Information). These results may be due to CDC20 and CDH1 being coactivators of APC/C and their similar protein structures.

### 2.2 CDC20 Deficiency Upregulates Protein Stability of PBRM1

CDC20 plays an important role in mitosis and its protein levels fluctuate during the cell cycle.^[^ ^31^, ^32^ ^]^ The PBRM1 protein abundance fluctuated during the cell cycle in cells synchronized by double thymidine treatment, displaying a dramatic reduction when CDC20 was most active (Figure 1H,I; Figure S1F,G, Supporting Information). Consistent with this finding, an inverse correlation between CDC20 activity and the abundance of PBRM1 was also observed in cells synchronized by nocodazole (Figure 1J,K) and serum starvation block (Figure 1L,M; Figure S1H,I, Supporting Information).

Previous results demonstrated that paclitaxel (Taxol) and nocodazole can induce G2/M arrest and activate SAC to suppress CDC20. Thus, nocodazole and taxol were used to block the cell cycle, and PBRM1 protein level was upregulated while CDC20 protein level was decreased (Figure S1J,K, Supporting Information). Furthermore, we found that CDC20 deficiency resulted in the upregulation of PBRM1 as well as other identified CDC20 substrates, including Cyclin B1 and p21 (Figure 1N,O), whereas CDH1 deficiency had no effect on PBRM1 (Figure S1L, Supporting Information). In keeping with these findings, depletion of CDC20 resulted in a rather stable pattern of PBRM1 expression throughout the cell cycle (Figure 1P,Q; Figure S1M,N, Supporting Information). Meanwhile, the half‐life of PBRM1 was remarkably prolonged in the CDC20 deficiency group (Figure 1R,S; Figure S1O,P, Supporting Information), whereas CDH1 deficiency did not affect the half‐life of PBRM1 (Figure S1Q,R, Supporting Information). In the PBRM1 mutant protein, the half‐life of 35RLA and B‐RLA were significantly prolonged when treated with CHX (Figure 1T,U). Furthermore, to investigate the relationship between PBRM1 and CDC20 in RCC patients, we collected 91 tumor tissues from patients in our hospital, and immunohistochemistry was performed to detect the expression of PBRM1 and CDC20 by using IHC score calculations. These results confirmed that the expression of PBRM1 was negatively correlated with CDC20 expression (Figure 1V,W). Collectively, these results indicate that CDC20 negatively regulated the protein stability of PBRM1.

### 2.3 APC/C^CDC20^ Functions as E3 Ubiquitin Ligase and Promotes K27 Ubiquitination of PBRM1

**Figure 2:**
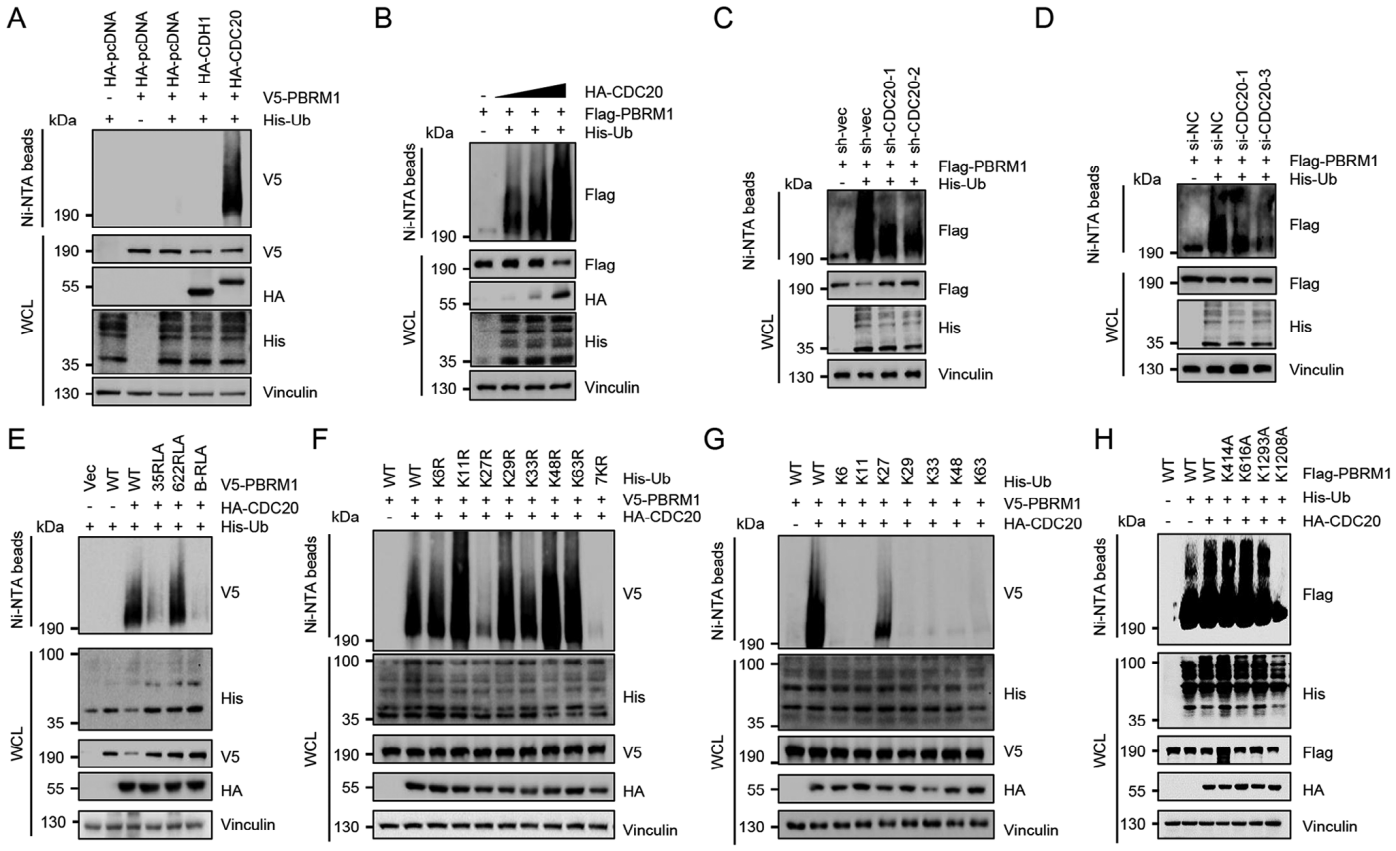
CDC20 catalyzes the Lys27‐linked ubiquitination of PBRM1. A) Immunoblotting (IB) analysis of WCL and Ni‐NTA pull‐down lysates derived from 293T cells transfected with indicated plasmids. B) IB analysis of WCL and Ni‐NTA pull‐down lysates derived from 293T cells transfected with Flag‐PBRM1, HA‐CDC20, and His‐Ub plasmids. C) IB analysis of WCL and Ni‐NTA pull‐down lysates derived from WT and CDC20 knockdown 293T cells transfected with Flag‐PBRM1 and His‐Ub plasmids. D) IB analysis of WCL and Ni‐NTA pull‐down lysates derived from 293T cells transfected with siCDC20, Flag‐PBRM1 and His‐Ub plasmids. NC, negative control. E) IB analysis of WCL and Ni‐NTA pull‐down lysates derived from WT and CDC20 knockdown 293T cells transfected with His‐Ub, HA‐CDC20, and indicated constructs of V5‐PBRM1 plasmids. F) IB analysis of WCL and Ni‐NTA pull‐down lysates derived from 293T cells transfected with HA‐CDC20, V5‐PBRM1 and indicated KR (Lys to Arg) ubiquitin mutants. G) IB analysis of WCL and Ni‐NTA pull‐down lysates derived from 293T cells transfected with HA‐CDC20, V5‐PBRM1 and K‐only ubiquitin mutants. H) IB analysis of WCL and Ni‐NTA pull‐down lysates derived from WT and CDC20 knockdown 293T cells transfected with His‐Ub, HA‐CDC20 and indicated constructs of Flag‐PBRM1 plasmids.

As a key regulator of mitosis and co‐activator of the E3 ubiquitin ligase APC/C, APC/C^CDC20^ might regulate the stability of PBRM1 by mediating its ubiquitination. To verify our hypothesis, the Ni‐NTA protein purification system was used to validate the ubiquitination of PBRM1 promoted by CDC20. Indeed, ubiquitination assays showed that CDC20, rather than CDH1, could increase PBRM1 protein ubiquitination (**Figure** **2**A). Moreover, CDC20 overexpression upregulated PBRM1 ubiquitination, whereas CDC20 knockdown decreased PBRM1 ubiquitination (Figure 2B–D). We further found that ubiquitination of PBRM1 was remarkably decreased in the 35RLA and B‐RLA mutants, but not in the 622RLA mutant, indicating that the promotional effect of CDC20 on PBRM1 ubiquitination was up to the^[^ ^35^ ^]^ RRRL^[^ ^38^ ^]^ domain at position 35 of PBRM1 (Figure 2E). To further evaluate the ubiquitination mechanism of PBRM1, we constructed different ubiquitin‐independent expression plasmids (K6, K11, K27, K29, K33, K48, and K63) and different ubiquitin mutant plasmids (K6R, K11R, K27R, K29R, K33R, K48R, K63R, and 7KR). Further experiments found that CDC20 promoted K27‐linked polyubiquitination of PBRM1 (Figure 2F,G). To determine the lysine residue(s) of PBRM1 ubiquitinated by CDC20, we applied mass spectrometry analysis of 293T cells transfected with Flag‐tagged PBRM1 in combination with HA‐tagged CDC20 and ubiquitin. Ubiquitination at lysine residues 414, 616, 1208, and 1293 in PBRM1 was detected by mass spectrometry (Figure S2A–D, Supporting Information). We then mutated the major individual lysine residues to alanine (K414A, K616A, K1208A and K1293A), and we found that mutation of K1208 (but not K414A, K616A or K1293A) decreased CDC20‐mediated polyubiquitination of PBRM1 (Figure 2H; Figure S2E, Supporting Information). Thus, we identified the formation of a polyubiquitin chain on PBRM1 K1208 mediated by CDC20. Consistent with this finding, compared with PBRM1 WT, the PBRM1 K1208 mutant was resistant to CDC20‐mediated degradation (Figure S2F–I, Supporting Information).

### 2.4 CDC20 Promotes p62‐Mediated Selective Autophagic Degradation of PBRM1

**Figure 3:**
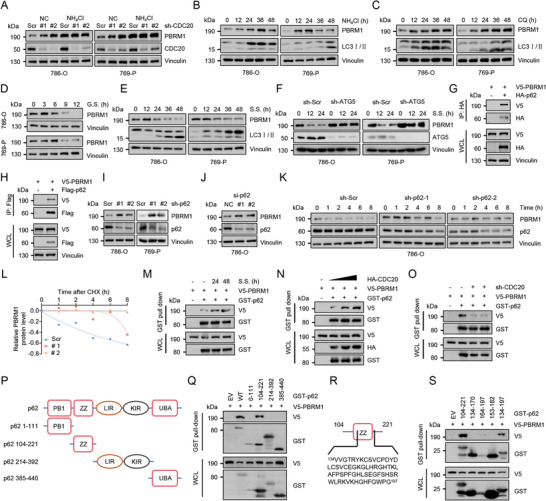
Autophagy selectively degrades PBRM1 via the autophagic receptor p62 in a CDC20‐dependent manner. A) IB analysis of WCL lysates derived from WT or CDC20 knockdown 786‐O and 769‐P cells treated with NH_4_Cl (20 mM, 24 h) before harvesting. B) IB analysis of WCL lysates derived from 786‐O and 769‐P cells treated with NH_4_Cl (20 mM) for indicated hours. C) IB analysis of WCL lysates derived from 786‐O and 769‐P cells treated with chloroquine (CQ, 50 µM) for indicated hours. D) IB analysis of WCL lysates derived from 786‐O and 769‐P cells treated with glucose starvation (G.S.) for indicated hours. E) IB analysis of WCL lysates derived from 786‐O and 769‐P cells treated with serum starvation (S.S.) for indicated hours. F) IB analysis of WCL lysates derived from wild‐type (WT) or ATG5 knockdown 786‐O and 769‐P cells treated with serum starvation (S.S.) for indicated hours before harvesting. G) IB analysis of WCL and anti‐HA immunoprecipitates derived from 293T cells transfected with HA‐p62 and V5‐PBRM1. H) IB analysis of WCL and anti‐Flag immunoprecipitates derived from 293T cells transfected with Flag‐p62 and V5‐PBRM1. I) IB analysis of WCL lysates derived from shScr or p62 knockdown 786‐O and 769‐P cells using shRNA. J) IB analysis of WCL lysates derived from NC or p62 knockdown 786‐O cells using siRNA. NC, negative control. K,L) Immunoblotting analysis (K) and quantification (L) of cell lysates of WT and p62 knockdown 786‐O cells treated with cycloheximide (CHX, 20 µg ml^−1^) at indicated time points. M) IB analysis of WCL and GST‐pull‐down products derived from 293T cells transfected with GST‐p62 and V5‐PBRM1. 24 h post transfection, cells were treated with serum starvation (S.S.) for 24 h before harvesting the cells. N) IB analysis of WCL and GST‐pull‐down products derived from 293T cells transfected with GST‐p62, HA‐CDC20 and V5‐PBRM1. O) IB analysis of WCL and GST‐pull‐down products derived from WT and CDC20 knockdown 293T cells transfected with GST‐p62 and V5‐PBRM1. P) Schematic representation of truncated constructs of p62 for mapping the interaction domain with PBRM1. Q) IB analysis of WCL and GST‐pull‐down products derived from 293T cells transfected with V5‐PBRM1 and indicated constructs of GST‐p62 plasmids. R) Schematic representation of truncated constructs of p62 for mapping the interaction domain with PBRM1. S) IB analysis of WCL and GST‐pull‐down products derived from 293T cells transfected with V5‐PBRM1 and indicated constructs of GST‐p62 plasmids.

To distinguish which degradation system dominantly regulates the degradation of PBRM1 promoted by CDC20, we further examined the protein stability of PBRM1 using MG132, which inhibits ubiquitin proteasome function, and ammonium chloride (NH_4_Cl), an inhibitor of autophagy. We found that NH_4_Cl increased the protein level of PBRM1 and attenuated CDC20 knockdown induced PBRM1 upregulation (**Figure** **3**A; Figure S3A, Supporting Information), indicating that PBRM1 protein stability was mostly controlled by the autophagy‐lysosome pathway. The protein level of PBRM1 was upregulated when autophagy was gradually inhibited by NH_4_Cl or chloroquine (CQ, Figure 3B,C), two well‐known autophagy inhibitors. Furthermore, we observed that autophagic inducer, glucose or serum starvation, decreased the protein level of PBRM1 (Figure 3D,E). In addition, PBRM1 was upregulated in ATG5 knockdown (KD) cells with or without serum starvation treatment (Figure 3F). Taken together, these results indicated that CDC20 promoted PBRM1 ubiquitination and degradation via autophagy.

To further analyze the mechanism by which PBRM1 is degraded by the autophagy‐lysosome pathway, we examined whether p62, a cargo receptor deliver cargoes to the autophagosome for selective degradation, participates in CDC20‐mediated PBRM1 stabilization. Our result demonstrated that PBRM1 interacted with p62 (Figure 3G,H) rather than HSC70 chaperone protein (Figure S3B, Supporting Information), confirming that PBRM1 was degraded by selective autophagy mediated by p62 rather than by chaperone‐mediated autophagy (CMA). Moreover, knockdown of p62 upregulated the protein expression of PBRM1 in 786‐O and 769‐P cells (Figure 3I,J), and the half‐life of PBRM1 was prolonged in the p62 deficiency group (Figure 3K,L; Figure S3C,D, Supporting Information). We also found that the association between PBRM1 and p62 increased considerably in response to serum starvation treatment (Figure 3M). Meanwhile, we found that CDC20 enhanced the interaction between PBRM1 and p62 (Figure 3N,O), indicating that CDC20 promoted autophagic degradation of PBRM1 through assisting PBRM1‐p62 interaction. To further confirm the specific regions of interaction between p62 and PBRM1, we constructed truncated plasmids with different p62 domains (Figure 3P). Further Co‐IP assays revealed the involvement of the region (104‐221) for the interaction with PBRM1, but not to other domains (Figure 3Q). Similarly, we constructed truncated plasmids within p62 104–221 and abbreviated the domain of p62 interacting with PBRM1 within 134–197 (Figure 3R,S). In conclusion, our results revealed that CDC20 promotes PBRM1 degradation through p62‐mediated selective autophagy.

### 2.5 Inhibition of PBRM1 Sensitizes RCC to Immunotherapy Through Regulating Macrophage‐Associated Chemokines and Increasing M1 Macrophage Infiltration

**Figure 4:**
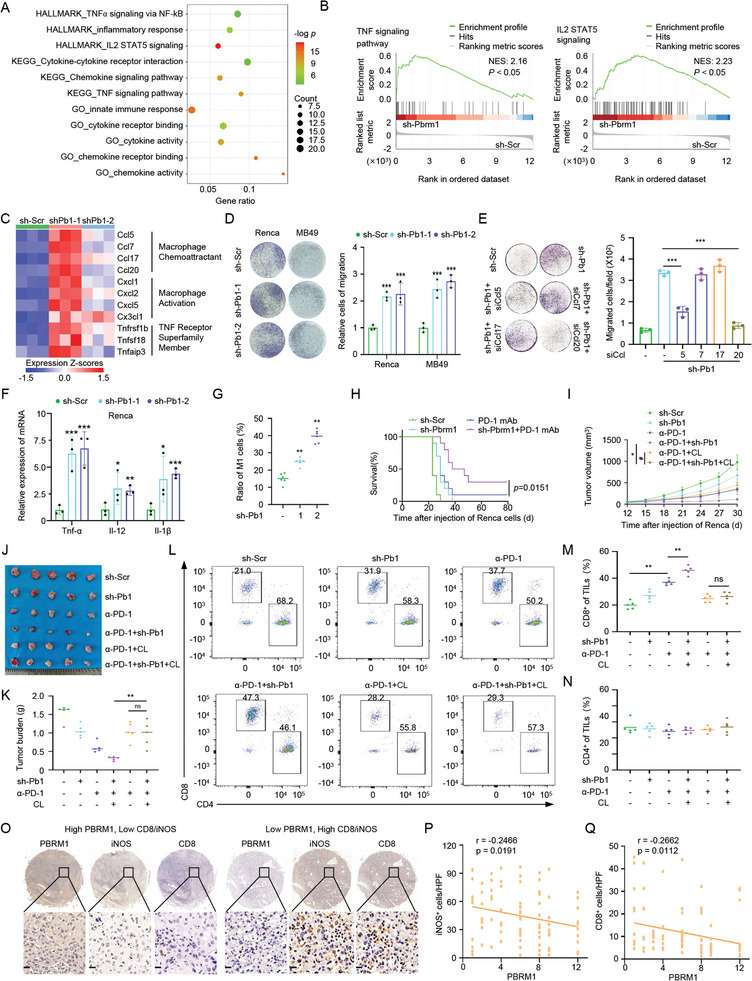
Inhibition of PBRM1 enhanced efficacy of PD‐1 blockade through regulating macrophage‐associated chemokines and increasing M1 macrophage infiltration. A) Immune‐related pathway enrichment analysis of wild‐type (WT) and PBRM1 knockdown Renca cells. *n* = 3. B) Gene set enrichment plots of TNF signaling pathway and IL2‐STAT5 signaling pathways according to RNA‐seq data in Figure 4A. C) Immune‐related differentially expressed genes (DEGs) for wild‐type (WT) and PBRM1 knockdown Renca cells according to RNA‐seq data in Figure 4A. D) Photomicrographs of the migratory RAW264.7 following coculture with wild‐type (WT) or Pbrm1 knockdown cells for 24 h in 24‐well transwell lower chambers. E) Photomicrographs of the migratory RAW264.7 following coculture with indicated genes knockdown Renca cells for 24 h in 24‐well transwell lower chambers. F) TNFα, IL‐12, and IL‐1β mRNA expression of RAW264.7 after treatment with conditioned medium derived from wild‐type (WT) or Pbrm1 knockdown Renca cells was determined by qTR‐PCR. *n* = 3. G) Proportions of M1 macrophage infiltrated in wild‐type (WT) or Pbrm1 knockdown Renca syngeneic tumors. *n* = 6. H) Kaplan–Meier survival curves of Balb/c mice bearing Renca syngeneic tumor, according to tumor growth data in Figure S4F (Supporting Information). *n* = 10. I) Renca cells stably expressing shPBRM1 were subcutaneously injected into Balb/c mice to establish xenograft model and treated with anti‐PD‐1 mAb (2mg per mice, three times) or Clodronate liposome (0.5 mg per mice, three times). Statistical analysis of the tumor volumes which were measured every three days and plotted individually, *n* = 5 J) Tumor sizes in Balb/c mice bearing Renca renal carcinoma with indicated treatment. *n* = 5. K) Tumor burdens in Balb/c mice bearing Renca renal carcinoma with indicated treatment. *n* = 5. L–N) Representative dot plots and the proportions of CD4^+^ and CD8^+^ tumor‐infiltrating lymphocytes (TILs) in wild‐type (WT) and Pbrm1 knockdown Renca syngeneic tumors. *n* = 5. O–Q) Representative images and quantifications of primary lung carcinoma patient samples stained for PBRM1, iNOS and CD8 expression by IHC assay. Scale bar, 20 µm.

Previous study has confirmed that the mutation and loss of function of PBRM1 promote immune cell infiltration, which may affect the efficacy of immunotherapy for RCC.^[^ ^33^ ^]^ Thus, we aimed to evaluate the effects and regulatory mechanisms of wild‐type PBRM1 on immunotherapy of RCC. We first constructed PBRM1 knockdown cell lines using the shRNA (Figure S4A, Supporting Information) and performed RNA‐seq analysis. Enrichment analysis of transcriptome data suggested that immune‐related signaling pathways were significantly enriched in PBRM1 knockdown cells (**Figure** **4**A). Gene set enrichment analysis (GSEA) showed enrichment of up‐regulated genes in immune‐related pathways, including TNF signaling pathway and IL2‐STAT5 signaling pathways (Figure 4B). Moreover, data derived from RNA‐seq analysis revealed that downstream genes of immune‐related pathways, especially a variety of chemokines, were significantly up‐regulated after PBRM1 knockdown (Figure 4C). Given the pivotal roles of chemokines in macrophage recruitment and activation, we hypothesized that PBRM1 knockdown exerts immunomodulatory effects by regulating macrophage recruitment. The findings demonstrated that depletion of PBRM1 resulted in enhanced macrophage infiltration (Figure 4D). Furthermore, we found that knockdown of Ccl7 or Ccl17 exhibited negligible impact on macrophage recruitment, while knockdown of Ccl5 or Ccl20 significantly attenuated macrophage recruitment induced by PBRM1 depletion (Figure 4E). In addition, the transcription levels of the markers of M1 macrophages, TNFα, IL12, and IL1b, were significantly upregulated after co‐culturing with PBRM1 knockdown tumor cell culture medium (Figure 4F; Figure S4B, Supporting Information). Moreover, we observed the presence of infiltrating macrophages in *Pbrm1* WT or knockdown (KD) Renca xenograft tumors. The results indicated that PBRM1 knockdown could stimulate the infiltration and differentiation of M1 macrophages in vivo (Figure 4G; Figure S4C–E, Supporting Information). The functional activity loss of the PBRM1 mutation has been clinically validated to potentially enhance the efficacy of targeted therapy.^[^ ^2^, ^34^, ^35^ ^]^ To investigate the hypothesis that PBRM1 knockdown may impact immunotherapy sensitivity in ccRCC, we administered anti‐PD‐1 mAb to BALB/c mice with Renca cells expressing either wild‐type or knockdown Pbrm1. Our findings demonstrate that Pbrm1 knockdown suppressed tumor growth and improved survival rates when combined with PD‐1 monoclonal antibody treatment (Figure 4H; Figure S4F, Supporting Information). To investigate the role of macrophages in PBRM1 knockdown‐mediated enhancement of immunotherapeutic effects, we utilized clodronate liposomes to deplete tumor‐infiltrating macrophages and assessed tumor volume and burden. The results demonstrated that depletion of macrophages abolished the augmenting immunotherapeutic effects mediated by PBRM1 knockdown in vivo (Figure 4I–K and Tables S2 and S3, Supporting Information), highlighting the crucial involvement of macrophage recruitment in PBRM1‐mediated immune evasion. Consistently, silencing PBRM1 significantly increased CD8^+^ tumor‐infiltrating T lymphocytes (TILs), which could be reversed by clearance of macrophages (Figure 4L–N; Figure S4G, Supporting Information). Furthermore, immunohistochemistry was performed on microarrays of RCC samples to assess the expression levels of iNOS (a marker for M1 macrophages), CD8 (a marker for CD8^+^ T cells), and PBRM1. Our findings indicate a significant inverse correlation between PBRM1 expression and the infiltration of M1 macrophage cells (Rho  =  −0.3138, *P*  = 0.0026) as well as CD8^+^ T cells (Rho  =  −0.2662, *P*  =  0.0112, Figure 4O–Q). These results revealed that PBRM1 inhibition could promote the infiltration of M1 macrophages into RCC, thereby improving the efficacy of anti‐PD‐1 immunotherapy and prolonging the survival of tumor‐bearing mice.

### 2.6 Design of a Peptide Targeting PBRM1 Enhances the Infiltration of M1 Macrophages and Efficacy of Anti‐PD‐1 Immunotherapy in RCC

**Figure 5:**
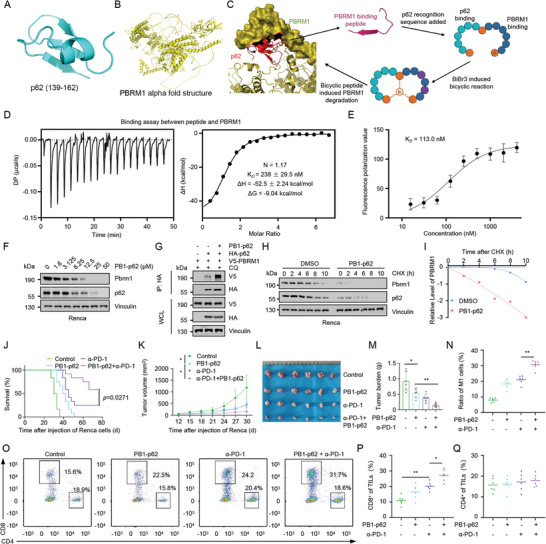
The PB1‐p62 drug effectively induces PBRM1 degradation and boost the impact of anti‐PD‐1 therapy. A) The protein crystal structure depicts amino acids 139 to 162 of p62 (obtained from PDB: 6MJ7). B) Alphafold prediction illustrates the full‐length structure of the PBRM1 protein. C) Schematic diagram and process of designing bicyclic peptide degradation drugs targeting PBRM1, based on the predicted protein sequence of p62 binding to PBRM1. D,E) Affinity detection of PB1‐p62 by ITC (D) and FP (E). F) IB analysis of PBRM1 in Renca cells after 24 h of treatment with the PB1‐p62 drug. G) IB analysis of WCL and anti‐HA immunoprecipitates derived from 293T cells transfected with HA‐p62 and V5‐PBRM1. 24 h post transfection, cells were treated with PB1‐p62 drug (5 µM) and chloroquine (CQ, 50 µM) for 24 h before harvesting the cells. H,I) IB analysis of cell lysates of Renca cells treated with PB1‐p62 drug (5 µM, 24 h) before treated with cycloheximide (CHX, 20 µgml^−1^) at indicated time points. J) Kaplan–Meier survival curves of Balb/c mice bearing Renca syngeneic tumor, according to tumor growth data in Figure S6F,G (Supporting Information). *n* = 12. K) Renca cells were subcutaneously injected into Balb/c mice to establish xenograft model and treated with anti‐PD‐1 mAb (2 mg per mice, three times) or PB1‐p62 (2 mg per mice, seven times). Statistical analysis of the tumor volumes which were measured every three days and plotted individually, *n* = 6. L) Tumor sizes in Balb/c mice bearing Renca renal carcinoma with indicated treatment. *n* = 6. M) Tumor burdens in Balb/c mice bearing Renca renal carcinoma with indicated treatment. *n* = 6. N) The proportions of M1 macrophage in Renca syngeneic tumors. *n* = 6. O–Q) Representative dot plots and the proportions of CD4^+^ and CD8^+^ tumor‐infiltrating lymphocytes (TILs) in Renca syngeneic tumors. *n* = 6.

We proposed that the regulation of PBRM1 by p62 may depend on both interaction and lysosomal sorting. To further analyze the interaction interface between p62 and PBRM1 at the structural level, we first examined the substrate recognition structure of p62 (**Figure** **5**A) and the protein structure of PBRM1 (Figure 5B). Using ZDOCK, we performed docking and interaction interface analysis of the p62‐PBRM1 protein complex. As shown in Figures S5A,B, Supporting Information, the substrate recognition structure of p62 interacts with PBRM1, with the most likely binding interface located at positions 1386–1451 of PBRM1. Co‐immunoprecipitation (Co‐IP) experiments at the cellular level also demonstrated the interaction between positions 139–162 of p62 and PBRM1 (Figure S5C, Supporting Information). Based on the interaction interface analysis between p62 and PBRM1, we designed novel degradation drugs targeting PBRM1 (Figure 5C). By identifying a peptide sequence that can bind to PBRM1 through its interaction interface with p62, we introduced the REE sequence at the N‐terminus of the recognition sequence signal of p62. This approach transforms a PBRM1 binding sequence into a PBRM1‐targeted degradation peptide sequence.

Peptide drugs offer advantages such as high affinity to target proteins and strong specificity. However, their clinical application is limited by their short half‐life and membrane penetration capabilities. The bicyclic peptide chemical method, which has garnered significant attention in recent years, can induce linear peptides to form bicyclic peptide structures, thus addressing the stability and membrane penetration issues of peptide drugs. In designing bicyclic peptides targeting PBRM1, we employed BiBr3 to induce the simultaneous reaction of three cysteines. We incorporated cysteines at the C‐terminus, N‐terminus, and the middle of the PBRM1 binding sequence and p62 recognition sequence, ultimately achieving the design of bicyclic peptide degradation drugs targeting PBRM1. We obtained linear PBRM1‐targeted degradation peptides through solid‐phase synthesis, which were subsequently converted into bicyclic peptide drugs using BiBr3 to induce bicyclic peptide formation. Mass spectrometry data confirmed that BiBr3 effectively facilitated the formation of bicyclic peptide structures (Figure S5D, Supporting Information). Upon completion of the bicyclic peptide synthesis, we employed ZDOCK to assess the binding affinity of both linear and bicyclic peptides to PBRM1. The results demonstrated that the bicyclic peptide PB1‐p62 exhibited stronger binding to PBRM1 compared to its linear counterpart. Specifically, both the ΔiG and ΔG values for PB1‐p62 were lower, indicating a more favorable binding interaction and higher affinity (Figure S5E,F, Supporting Information). The chemical structures of the linear and cyclic peptides are shown in Figure S5G,H (Supporting Information). The membrane‐penetrating ability of peptide drugs is critical for their efficacy. To evaluate this, we labeled PB1‐p62 with rhodamine and used flow cytometry to assess its cellular uptake. The flow cytometry results indicated that PB1‐p62 effectively penetrated the cell membrane and entered the cells within 6 h (Figure S5I, Supporting Information). Next, we evaluated the high affinity of the PB1‐p62 for PBRM1 using isothermal titration calorimetry (ITC) (Figure 5D) and fluorescence polarization (FP) (Figure 5E). The binding affinity between PB1‐p62 and the p62 protein was also measured by ITC (Figure S5J, Supporting Information). Serum stability experiments demonstrated that the bicyclic peptide significantly extended the half‐life of the peptide drug (Figure S5K, Supporting Information), thereby enhancing its potential for therapeutic applications.

As a result, the fusion constructs PB1‐p62 significantly promoted the degradation of PBRM1 (Figure 5F; Figure S5L–N, Supporting Information). Meanwhile, proteasome inhibitor MG132 could also exhibit the ability to enhance the protein level of PBRM1 (Figure S5O, Supporting Information), suggesting that PB1‐p62 facilitates the degradation of PBRM1 through autophagy and proteasome pathways. PB1‐p62 markedly enhanced the interaction between PBRM1 and p62 (Figure 5G) and decreased the half‐life of PBRM1 protein (Figure 5H,I; Figure S5P,Q, Supporting Information). To evaluate the druggability potential of PB1‐p62, we conducted in vivo toxicity assessments. The results revealed no significant alterations in alanine transaminase (ALT), aspartate transaminase (AST), creatinine (Cr), and blood urea nitrogen (BUN) levels between the PB1‐p62 peptide treatment groups and the control group (Figure S6A, Supporting Information). Histological examination of vital organs, including heart, liver, spleen, lung, and kidney, also demonstrated an absence of toxicity (Figure S6B, Supporting Information). Furthermore, our investigation into the impact of PB1‐p62 on tumor progression confirmed its dose‐dependent inhibitory effect on tumor growth (Figure S6C–E and Tables S4 and S5, Supporting Information). To verify the effect of PB1‐p62 on renal cancer immunotherapy, we performed Renca subcutaneous tumor formation experiments in BALB/c mice. PB1‐p62 in combination with the PD‐1 monoclonal antibody significantly prolonged the survival time of tumor‐bearing mice, suppressed tumor proliferation, and reduced tumor burden when compared with PD‐1 monoclonal antibody alone, suggesting that PB1‐p62 can enhance the efficacy of the PD‐1 monoclonal antibody (Figure 5J–M; Figure S6F,G and Tables S6 and S7, Supporting Information). In addition, we confirmed that PB1‐p62 in combination with the PD‐1 monoclonal antibody may enhance the therapeutic effect of the PD‐1 monoclonal antibody by increasing the infiltration of immune cells. The results indicated that PB1‐p62 increased the infiltration of M1 macrophages into mouse RCC tissues, whereas no significant difference was detected in the infiltration of M2 macrophages among the groups (Figure 5N; Figure S6H,I, Supporting Information). We also found that PB1‐p62 significantly improved the infiltration of CD8^+^ TILs in the subcutaneous tumors of mice induced by the PD‐1 monoclonal antibody (Figure 5O–Q). These findings together suggest that PB1‐p62 enhances the anti‐PD‐1 immunotherapy through both increasing the infiltration of M1 macrophages and CD8^+^ T cells.

## 3 Discussion

**Figure 6:**
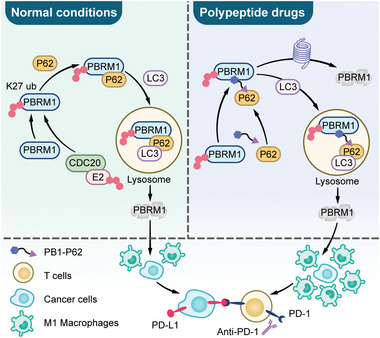
A schematic diagram to show CDC20‐mediated selective autophagy degradation of PBRM1 and regulates tumor immune evasion in renal cell carcinoma.

Somatic mutations of PBRM1, which are especially prevalent in clear cell renal cell carcinoma (ccRCC), always co‐occurred with deletion of the non‐mutated PBRM1 allele, consequently resulting in the loss of polybromo‐1 function. Previous studies have found that PBRM1 mutation was a promising biomarker for immunotherapy in ccRCC. Here we demonstrated that inhibition of wild‐type PBRM1 could also sensitizes RCC to immunotherapy through regulating macrophage‐associated chemokines and increasing M1 macrophage infiltration. Nonetheless, we aimed to improve the effects of immunotherapy through downregulating PBRM1 in *PBRM1* wild‐type RCC. In this study, we demonstrated that CDC20 interacts with PBRM1 and promotes K27 ubiquitination at the K1208 site, which weakens the stability of PBRM1. In particular, we validated that CDC20‐mediated PBRM1 degradation was via p62‐dependent selective autophagy. In addition, we synthesized a peptide to promote the degradation of PBRM1 by enhancing the interaction between PBRM1 and p62, providing a promising tumor therapeutic approach with PBRM1 inhibition and PD‐1 blockade (**Figure** **6**).

Many studies have demonstrated that the differentiation of macrophages and ratio of M1/M2 macrophages are related to tumor therapy, in which the M1 macrophage phenotype is classified to have an anti‐tumor effect and M2 has the opposite effect.^[^ ^36^, ^37^, ^38^ ^]^ We previously showed that the status of PBRM1 may be related to RCC immunotherapy.^[^ ^33^ ^]^ Our data demonstrate that alterations in PBRM1 particularly promote polarization of M1 macrophages, thereby inducing remodeling of the immune microenvironment and augmenting immunotherapeutic effects. Macrophages, as pivotal immune cells within the tumor microenvironment (TME), play intricate roles in driving tumor progression. M1 macrophage polarization has been confirmed to exert antineoplastic effects,^[^ ^39^ ^]^ which partly explains the reason why PBRM1 knockdown enhanced the response to immune checkpoint inhibitors. Moreover, our data confirmed that Ccl5 or Ccl20 act as the key chemokines involved in PBRM1 knockdown‐mediated macrophage recruitment, although the specific mechanism leading to Ccl5 or Ccl20 up‐regulation remains to be defined. Furthermore, we identified CDC20 as the upstream regulator of PBRM1, which provides a novel viewpoint for CDC20 to influence tumor immunity. In addition, the tissues of RCC patients indicated that lower level of PBRM1 was related to increased infiltration of M1 macrophages and CD8^+^ T cells. Thus, the results of our study demonstrated that PBRM1 may be a new target for RCC patients with wild‐type PBRM1 to improve the clinical efficacy of immunotherapy.

More importantly, we designed a peptide named PB1‐p62 to promote the autophagic degradation of PBRM1 by enhancing the interaction between PBRM1 and p62. A continuous amino acid sequence was truncated at the protein‐protein interaction interface between p62 and PBRM1 as the binding moiety of PBRM1. To achieve the targeted degradation of PBRM1, the p62 feature recognition REEE sequence^[^ ^40^ ^]^ was added to the N‐terminus of PBRM1 binding peptide and served as a p62 recognition signal. In addition, a series of assays were conducted to prove that PB1‐p62 was efficient in promoting the interaction between PBRM1 and p62 and enhancing the degradation of PBRM1. Our study further validated that PB1‐p62 improves immune efficacy by increasing the infiltration of M1 macrophages and T cells, which prolonged the survival of PBRM1 wild‐type tumor‐bearing mice. In fact, many therapeutic targets of cancer and other diseases are selective autophagy substrates, such as cGAS and PD‐L1, among others. Thus, it is of interest to develop novel lysosome‐targeting chimeras against these disease‐related proteins in future studies.

In summary, our work identifies CDC20 as a regulator of PBRM1 lysosomal degradation, providing new insight into the mechanisms that control the homeostasis of PBRM1. CDC20 regulates PBRM1 K27‐type ubiquitination and promotes p62‐mediated selective autophagic degradation of PBRM1. Thus, the rationalized design of chimeric peptides targeting the degradation process may greatly reduce the level of PBRM1 in renal cancer and improve the efficacy of immunotherapy. This will provide a new research direction for improving the immune efficacy of PBRM1 wild‐type patients in the clinic and greatly increase the possibility of targeted peptides in the clinical treatment of PBRM1 wild‐type patients.

## 4 Experimental Section

### Antibodies, Plasmids, and Reagents

Antibodies against PBRM1 (91 894), Vinculin (13 901), Cyclin D1 (2978), Cyclin B1 (12 231), p62 (5144), V5(13202), ATG5(12994), hemagglutinin (HA; 3724), and Flag (2368) were purchased from Cell Signaling Technology. CDC20 (10252‐1‐AP), CDH1 (16368‐1‐AP), His(66005‐1‐Ig), GST(10000‐0‐AP) and LC3(14600‐1‐AP) were purchased from Proteintech. Cyclin E (MA5‐14336) was purchased from Invitrogen. pCMV‐8 x His‐Ub (107392) and pcDNA6.2/N‐EmGFP‐PBRM1‐V5 (65387) were purchased from Addgene. pcDNA3.1‐PBRM1‐3xFlag was purchased from Biokeeper. pcDNA3‐HA‐CDC20, pcDNA3‐HA‐CDH1, pcDNA3‐HA‐Hsc70, pcDNA3‐HA‐p62, pcDNA3‐Flag‐p62, pCMV‐GST‐p62, and pCMV‐GST‐PBRM1 were constructed in our laboratory, and pcDNA6.2/N‐EmGFP‐PBRM1 35RLA, 622RLA, B‐RLA, and pCMV‐GST‐p62 domain plasmids were amplified and cloned according to standard protocols (KOD‐Plus‐Mutagenesis Kit; SMK‐101). Nocodazole (487928), double thymidine (T1895), and taxol (580555) were purchased from Sigma–Aldrich. Chloroquine (CQ; T8689), MG‐132 (T2154), NH_4_Cl (T64755), Cycloheximide (CHX; T1225) were purchased from Targetmol. Different mutants were generated by site‐directed mutagenesis PCR reaction using platinum PWO SuperYield DNA polymerase (Roche, Basel, Switzerland) according to the product manual.

### Cell Culture, Transfection, and Construction of Knockdown Cell Lines

The cell lines 786‐O, 769‐P, MB49, HEK293T, Raw264.7, and Renca used in this study were purchased from ATCC (Manassas, VA, USA) and provided by the Institute of Urology, First Affiliated Hospital of Xi’ an Jiaotong University. All cells were cultured in RPMI‐1640 or DMEM medium (Gibco) with 10% fetal bovine serum (Gibco) and maintained at 37 °C in 5% CO_2_. The cells were subjected to a double thymidine block assay, wherein they were incubated with 2 mM double thymidine for 18 h during the second round and then release. Cells synchronized with nocodazole‐arrest (100 ng mL^−1^, 18 h) and release were collected at the indicated time points.

PEI Prime linear polyethylenimine (PEI; 919 012) was purchased from Sigma–Aldrich and dissolved in DMSO, according to the standard protocol. PEI is suitable for gene delivery, and it was used for plasmid transfection in this study. Knockdown of PBRM1, CDC20, CDH1, and p62 was performed using shRNA, which were packaged with pSPAX2 and pMD2.G in HEK293T cells. All these components formed a lentiviral vector, which was collected to infect the target cell line. Puromycin (1 µg mL^−1^) was used to select the infected cells for 2 weeks, and the protein was extracted to validate target knockdown efficacy. All knockdown cell lines were cultured as described previously and used for assays within 6 months.

### Western Blot

Cells were washed 3 times with ice‐cold phosphate‐buffered saline (PBS). The appropriate RIPA or IP buffer was added, which was mixed with protease and phosphatase inhibitors in advance. After centrifugation, the cell lysate supernatant was collected and checked using the BCA qualification system. The appropriate protein‐loading buffer was added to the protein lysates and denatured in a boiling water bath for 5 min. Each sample contained 30 µg protein, which was loaded onto 8% or 10% SDS‐PAGE gels and transferred to a polyvinylidene fluoride (PVDF) membrane. Then, the PVDF membranes were immunoblotted in fresh milk for 1 h at room temperature. Primary antibodies of the targets were added to the PVDF membranes and incubated overnight at 4 °C. The cells were rinsed 3 times with TBST buffer, followed by incubation with peroxidase‐conjugated antibodies at room temperature for 1 h. After washing, the PVDF membranes were analyzed and visualized using an ECL system (Bio‐Rad, Hercules, CA, USA).

### Total RNA Extraction and Real‐Time RT‐PCR Analysis

Total cell RNA was extracted using TRIzol reagent (Invitrogen; Thermo Fisher Scientific) and the cDNA was synthesized using Primer Script RT reagent kit (Takara Bio). cDNA was diluted in SYBR Green Master Mix (Takara Bio) and then real‐time RT‐PCR was conducted following cycling conditions: 95 °C for 15 s, 55 °C for 30 s, and 72 °C for 30 s. Relative mRNA levels were evaluated using the 2^−ΔΔ^ CT method. The primer sequences were listed in Table S1 (Supporting Information).

### Immunoprecipitation and Co‐Immunoprecipitation Assay

Suitable protease and phosphatase inhibitors were added to the IP lysis buffer (50 mM Tris‐HCl [pH 7.4], 120 mM NaCl, 5 mM EDTA, and 0.5% NP‐40) was used to lyse cells, and the proteins were extracted as described previously. According to the experimental design, 0.5 mL protein solution (1 mg) was collected from the top of each sample, and 10 µL primary targets or Flag/HA antibody coupled with agarose particles was added to each sample. After sealing, the samples were incubated in a refrigerator for 4–6 h. At the end of the reaction, the EP tube was removed, the supernatant was discarded after centrifugation at 15 000 × *g* for 30 s, and 1 mL of precooled IP lysate was added and used several times for rinsing. An appropriate amount of IP lysate containing 1× loading buffer was added to each group of samples and placed in a boiling water bath for ≈10 min and then removed and placed in a −20 °C refrigerator. The experimental results were by performing the western blotting experiment the next day.

### GST Pull‐Down Assay

In our study, pGEX‐4T‐1 (Addgene, 129 567), pGEX‐4T‐1‐GST‐PBRM1, and pGEX‐4T‐1‐GST‐CDC20/CDH1 vectors were constructed and expressed in *Escherichia coli* BL21. The transfected bacteria were collected, and a suitable IP buffer was added for ultrasonic lysis. Targets with GST tags were purified using Glutathione Sepharose 4B beads (17 075 605; GE Healthcare). pcDNA3‐HA‐CDC20/CDH1 was expressed in HEK293T cells, and the proteins were purified using HA‐tag beads (Sigma–Aldrich, A2220). Purified GST‐PBRM1 and HA‐CDC20/CDH1 were incubated together at 4 °C for GST pull‐down assays. The system should spin softly for no more than 3 h. Finally, the beads were washed twice with IP buffer and maintained at 95 °C for 5 min, and the proteins were collected and analyzed with western blotting, as described previously.

### Ni‐NTA Pull‐Down Assay

Flag‐PBRM1/GFP‐PBRM1‐V5, HA vector, HA‐CDC20, and His‐Ub were transfected and expressed in HEK293T cells. After washing with pre‐cooled PBS, the appropriate cells were lysed with IP buffer for the whole cell lysis group. The remaining cells were lysed with buffer A (6 M guanidine‐HCl, 0.1 M Na_2_HPO_4_/NaH_2_PO_4_, and 10 mM imidazole [pH 8.0]). After sonication for 10 s, the proteins were collected by centrifugation and incubated with the appropriate nickel‐nitrilotriacetic acid (Ni‐NTA) beads (Qiagen) for 2–3 h at room temperature. Then, the Ni‐NTA beads were washed 2 times with buffer A and buffer A/TI (1:3 of volume buffer A and buffer TI) and once with buffer TI (25 mM Tris‐HCl, 20 mM imidazole [pH 6.8]). Finally, a suitable IP buffer and protein‐loading marker were added to the washed beads, which were then placed in boiling water for 5 min to denature the proteins. The pull‐down proteins were separated and analyzed with western blotting.

### Flow Cytometry Analysis (FACS)

Flow cytometry was used to analyze immune cells infiltrated in TME. Briefly, immune cells were extracted from xenograft tumors, and washed with MACS buffer (PBS, 2%FBS, 1 mM EDTA). Surface staining was performed by adding the indicated antibodies to the cell suspension: anti‐CD8 (Biolegend: 126 614), anti‐CD4 (Biolegend: 100 432), and anti‐CD3 (Biolegend: 100 206), F4/80 (BioLegend: 157 304), CD11c (BioLegend: 117 326), CD206(BioLegend: 141 708). After 30 min incubation, cells were washed with MACS buffer and analyzed on BD Fortessa flow cytometer. The results were analyzed with Flowjo software.

### Immunohistochemical Assay

Ninety‐four RCC specimens were obtained from the First Affiliated Hospital of Xi'an Jiaotong University (Shaanxi, China). Informed consent was obtained for the human RCC specimens and the usage of these specimens was approved by the Ethic Committee of First Affiliated Hospital of Xi'an Jiaotong University (NO. LLSBPJ‐2023‐059). The tumor tissues from the RCC patients were fixed with 10% formaldehyde for 24 h, embedded in paraffin, and cut into 5‐µm‐thick sections. After deparaffinization, rehydration, endogenous peroxidase blocking, and antigen retrieval, the sections were blocked with 5% BSA for 0.5 h and incubated with primary antibodies at 4 °C overnight. The next day, the tumor tissue sections were incubated with secondary antibodies for 1 h and visualized using a DAB kit, according to the manufacturer's instructions. Photographs were obtained and analyses were performed using a microscope (Olympus Optical Co., Tokyo, Japan). Three random fields (×100) of each tumor tissue in each section were photographed and scored for further analysis. The grading of staining intensity was as follows: no staining (score 0), light brownish‐yellow (score 1), brownish‐yellow (score 2), and dark brownish‐brown (score 3). The grading of positive cell count was as follows: less than 25% positive cells were graded as score 1, 26% to 50% were graded as score 2, 51% to 75% were graded as score 3, and ≥75% were graded as score4. The final score was calculated by multiplying the staining intensity score with the positive cell count score.

### Xenograft Animal Model for RCC

All animal experiments were authorized by the Health Science Center (HSC) of Xi'an Jiaotong University (NO. XJTUAE2023‐285), and the animals were killed according to standard ethical guidelines. Forty BALB/c mice were purchased for each experiment, and these mice were 5 weeks of age and weighed ≈20 g. Renca cells were harvested and suspended in serum‐free medium containing Matrigel, and 200 µL of Renca cells (1 × 10^6^ cells) was subcutaneously injected into the right lower limbs of each mouse. After the mice were fed for 1 week and the tumors grew to ≈100–150 mm^3^ in size, they were randomly divided into 4 groups, and each group was intraperitoneally injected with placebo, PB1‐p62 (2 mg kg^−1^, or knockdown of PBRM1), or anti‐PD‐1 monoclonal antibody with or without PB1‐p62 (or knockdown of PBRM1) every 3 days. The anti‐PD‐1 monoclonal antibody (BE0146), named InVivoMAb anti‐mouse PD‐1 (CD279), was purchased from Bio X Cell and used according to the manufacturer's instructions. The longest and shortest tumor diameters were measured at the same frequency. After 3 weeks, the tumors were harvested and ground to separate and extract immune cells, and the results were analyzed using flow cytometry. Survival assays were performed until the mice died or the tumor reached its setting volume. Tumor volume was calculated using the following formula: tumor volume = (length × width^2^) × 0.5. We also assessed it by tumor inhibitory rate (TIR). TIR was calculated as follows: TIR  =  [1 − (Vt / Vc)] × 100% or TIR  =  [1 − (Wt / Wc)] × 100%, where Vt is the final mean tumor volume of the tested groups, Vc is the final mean tumor volume of the control group, Wt is the final mean tumor weight of the tested groups, and Wc is the final mean tumor weight of the control group.

### Statistical Analysis

All experiments were performed at least 3 times, and the data were represented as mean ± standard deviation (SD) values. Differences between groups were analyzed using one‐way ANOVA. Only 2 groups were compared with Student's *t*‐test, and the Mann–Whitney *U* test was used for IHC analysis. ImageJ software (NIH, Bethesda, MD, USA) was used to quantify the densities of the bands in the western blot. *p* < 0.05 was used as the statistically significant standard in all cases.

## Conflict of Interest

The authors declare no conflict of interest.

## Supporting information



Supporting Information

## Data Availability

The data that support the findings of this study are available from the corresponding author upon reasonable request.

## References

[1] L. Bukavina , K. Bensalah , F. Bray , M. Carlo , B. Challacombe , J. A. Karam , W. Kassouf , T. Mitchell , R. Montironi , T. O'Brien , V. Panebianco , G. Scelo , B. Shuch , H. van Poppel , C. D. Blosser , S. P. Psutka , Eur. Urol. 2022, 82, 529. 36100483 10.1016/j.eururo.2022.08.019

[2] I. Varela , P. Tarpey , K. Raine , D. Huang , C. K. Ong , P. Stephens , H. Davies , D. Jones , M.‐L. Lin , J. Teague , G. Bignell , A. Butler , J. Cho , G. L. Dalgliesh , D. Galappaththige , C. Greenman , C. Hardy , M. Jia , C. Latimer , K. W. Lau , J. Marshall , S. McLaren , A. Menzies , L. Mudie , L. Stebbings , D. A. Largaespada , L. F. A. Wessels , S. Richard , R. J. Kahnoski , J. Anema , et al., Nature 2011, 469, 539. 21248752 10.1038/nature09639 PMC3030920

[3] X. Yao , J. H. Hong , A. M. Nargund , M. S. W. Ng , H. L. Heng , Z. Li , P. Guan , M. Sugiura , P. L. Chu , L. C. Wang , X. Ye , J. Qu , X. Y. Kwek , J. C. T. Lim , W. F. Ooi , J. Koh , Z. Wang , Y.‐F. Pan , Y. S. Ong , K.‐Y. Tan , J. Y. Goh , S. R. Ng , L. Pignata , D. Huang , A. Lezhava , S. T. Tay , M. Lee , X. H. Yeo , W. L. Tam , S. Y. Rha , et al., Nat. Cell Biol. 2023, 25, 765. 37095322 10.1038/s41556-023-01122-y

[4] L. Meng , Cells 2023, 13.

[5] D. Pan , A. Kobayashi , Science 2018, 359, 770. 29301958 10.1126/science.aao1710 PMC5953516

[6] D. A. Braun , Y. Ishii , A. M. Walsh , E. M. Van Allen , C. J. Wu , S. A. Shukla , T. K. Choueiri , JAMA Oncol. 2019, 5, 1631. 31486842 10.1001/jamaoncol.2019.3158 PMC6735411

[7] S. Bruno , A. G. Luserna di Rora , R. Napolitano , S. Soverini , G. Martinelli , G. Simonetti , J. Exp. Clin. Cancer Res. 2022, 41, 159. 35490245 10.1186/s13046-022-02363-9 PMC9055704

[8] L. F. Chang , Z. Zhang , J. Yang , S. H. McLaughlin , D. Barford , Nature 2014, 513, 388. 25043029 10.1038/nature13543 PMC4456660

[9] H. Yu , Mol. Cell 2007, 27, 3. 17612486 10.1016/j.molcel.2007.06.009

[10] C. Doornbos , R. Roepman , Cell. Mol. Life Sci. 2021, 78, 4955. 33860332 10.1007/s00018-021-03827-5 PMC8233288

[11] S. Okuda , M. Sato , S. Kato , S. Nagashima , R. Inatome , S. Yanagi , T. Fukuda , J. Biol. Chem. 2021, 297, 100986. 34298015 10.1016/j.jbc.2021.100986 PMC8353494

[12] N. Sanz‐Gómez , I. de Pedro , B. Ortigosa , D. Santamaría , M. Malumbres , Cell Death Differ. 2020, 27, 2451. 32080348 10.1038/s41418-020-0515-2 PMC7370216

[13] S. B. Lee , J. J. Kim , H.‐J. Nam , B. Gao , P. Yin , B. Qin , S.‐Y. Yi , H. Ham , D. Evans , S.‐H. Kim , J. Zhang , M. Deng , T. Liu , H. Zhang , D. D. Billadeau , L. Wang , E. Giaime , J. Shen , Y.‐P. Pang , J. Jen , J. M. van Deursen , Z. Lou , Mol. Cell 2015, 60, 21. 26387737 10.1016/j.molcel.2015.08.011 PMC4592523

[14] H. Fujita , T. Sasaki , T. Miyamoto , Aging Cell 2020, 19, e13251. 33094908 10.1111/acel.13251 PMC7681047

[15] Y.‐P. Xie , S. Lai , Q.‐Y. Lin , X. Xie , J.‐W. Liao , H.‐X. Wang , C. Tian , H.‐H. Li , Theranostics 2018, 8, 5995. 30613277 10.7150/thno.27706 PMC6299438

[16] Q. Gu , F. Li , S. Ge , F. Zhang , R. Jia , X. Fan , Mol. Ther. Oncol. 2020, 17, 94. 10.1016/j.omto.2020.03.015 PMC7163048 32322666

[17] A. C. Chun , K. H. Kok , D. Y. Jin , Cell Cycle 2013, 12, 365. 23287467 10.4161/cc.23214 PMC3575465

[18] M. V. Hadjihannas , D. B. Bernkopf , M. Brückner , J. Behrens , EMBO Rep. 2012, 13, 347. 22322943 10.1038/embor.2012.12 PMC3321148

[19] D. D. Mao , A. D. Gujar , T. Mahlokozera , I. Chen , Y. Pan , J. Luo , T. Brost , E. A. Thompson , A. Turski , E. C. Leuthardt , G. P. Dunn , M. R. Chicoine , K. M. Rich , J. L. Dowling , G. J. Zipfel , R. G. Dacey , S. Achilefu , D. D. Tran , H. Yano , A. H. Kim , Cell Rep. 2015, 11, 1809. 26074073 10.1016/j.celrep.2015.05.027 PMC4481182

[20] Y. Gao , P. Wen , B. Chen , G. Hu , L. Wu , A. Xu , G. Zhao , Int. J. Mol. Sci. 2020, 21. 10.3390/ijms21186692 PMC7555290 32932732

[21] Z. Chen , Y. Yu , D. Fu , Z. Li , X. Niu , M. Liao , S. Lu , Cell Biochem. Funct. 2010, 28, 249. 20517887 10.1002/cbf.1634

[22] J. M. Eichhorn , N. Sakurikar , S. E. Alford , R. Chu , T. C. Chambers , Cell Death Dis. 2013, 4, e834. 24091677 10.1038/cddis.2013.360 PMC3824670

[23] L. Wan , M. Tan , J. Yang , H. Inuzuka , X. Dai , T. Wu , J. Liu , S. Shaik , G. Chen , J. Deng , M. Malumbres , A. Letai , M. W. Kirschner , Y. Sun , W. Wei , Dev. Cell 2014, 29, 377. 24871945 10.1016/j.devcel.2014.04.022 PMC4081014

[24] X. Lai , Y.‐K. Wu , G.‐Q. Hong , J.‐K. Li , Q. Luo , J. Yuan , G.‐H. Dai , S.‐Q. Liu , H.‐G. Feng , J. Immunol. Res. 2022, 2022, 9117205. 35402624 10.1155/2022/9117205 PMC8986430

[25] T. Si , Z. Huang , Y. Jiang , A. Walker‐Jacobs , S. Gill , R. Hegarty , M. Hamza , S. E. Khorsandi , W. Jassem , N. Heaton , Y. Ma , Front Oncol. 2021, 11, 738841. 34660300 10.3389/fonc.2021.738841 PMC8515852

[26] J. Shi , Y. Chen , X. Gu , X. Wang , J. Liu , X. Chen , Contrast Media Mol. Imaging 2022, 2022, 7727539. 35800227 10.1155/2022/7727539 PMC9200543

[27] N. Mizushima , M. Komatsu , Cell 2011, 147, 728. 22078875 10.1016/j.cell.2011.10.026

[28] V. Rogov , V. Dötsch , T. Johansen , V. Kirkin , Mol. Cell 2014, 53, 167. 24462201 10.1016/j.molcel.2013.12.014

[29] S. Shaid , C. H. Brandts , H. Serve , I. Dikic , Cell Death Differ. 2013, 20, 21. 22722335 10.1038/cdd.2012.72 PMC3524631

[30] T. Lamark , S. Svenning , T. Johansen , Essays Biochem. 2017, 61, 609. 29233872 10.1042/EBC20170035

[31] M. Kapanidou , N. L. Curtis , V. M. Bolanos‐Garcia , Trends Biochem. Sci. 2017, 42, 193. 28202332 10.1016/j.tibs.2016.12.001

[32] Z. Wang , Methods Mol. Biol. 2022, 3, 277. 10.1007/978-1-0716-2221-6_19 35635710

[33] T. Liu , Q. Xia , H. Zhang , Z. Wang , W. Yang , X. Gu , T. Hou , Y. Chen , X. Pei , G. Zhu , D. He , L. Li , S. Xu , Aging 2020, 12, 21809. 33177244 10.18632/aging.103999 PMC7695370

[34] D. Miao , C. A. Margolis , Science 2018, 359, 801. 29301960 10.1126/science.aan5951 PMC6035749

[35] L. Carril‐Ajuria , M. Santos , J. M. Roldán‐Romero , C. Rodriguez‐Antona , G. Velasco , Cancers 2019, 12, 16. 31861590 10.3390/cancers12010016 PMC7016957

[36] J. L. Guerriero , Trends Mol. Med. 2018, 24, 472. 29655673 10.1016/j.molmed.2018.03.006 PMC5927840

[37] A. Puig‐Kröger , E. Sierra‐Filardi , A. Domínguez‐Soto , R. Samaniego , M. T. Corcuera , F. Gómez‐Aguado , M. Ratnam , P. Sánchez‐Mateos , A. L. Corbí , Cancer Res. 2009, 69, 9395. 19951991 10.1158/0008-5472.CAN-09-2050

[38] P. Italiani , D. Boraschi , Front. Immunol. 2014, 5, 514. 25368618 10.3389/fimmu.2014.00514 PMC4201108

[39] Z. Duan , Y. Luo , Signal Transduct. Target Ther. 2021, 6, 127. 33767177 10.1038/s41392-021-00506-6 PMC7994399

[40] Y. Zhang , S. R. Mun , Nat. Commun. 2018, 9, 4373. 30349045 10.1038/s41467-018-06878-8 PMC6197226

