# Supplementary material for: CDC20‐Mediated Selective Autophagy Degradation of PBRM1 Affects Immunotherapy for Renal Cell Carcinoma
Source: Adv Sci (Weinh). 2024 Dec 10;12(5):2412967. doi: 10.1002/advs.202412967 (PMC11791976; doi:10.1002/advs.202412967)
Supplement: Supplementary file 1 — Supporting Information [file ADVS-12-2412967-s001.docx]

Supplementary Figures for

**CDC20-mediated** **Selective Autophagy Degradation of PBRM1 Affects Immunotherapy for Renal Cell Carcinoma**

Yizeng Fan^1,2,3,4^, Weichao Dan^1,2,3,4^, Taotao Que^1,2,3,4^, Yi Wei^1,2,3^, Bo Liu^1,2,3^, Zixi Wang^1,2,3^, Yulin Zhang^1,2,3^, Yuzhao Wang^1,2,3^, Tianjie Liu^1,2,3^, Yanxin Zhuang^1,2,3^, Mengxing Li^1,2,3^, Chendong Guo^1,2,3^, Jin Zeng^1,2,3^, Bohan Ma^1,2,3,*^ and Lei Li^1,2,3,*^

^1^ Department of Urology, The First Affiliated Hospital of Xi'an Jiaotong University, Xi'an, Shaanxi 710061, P. R. China

^2^ Key Laboratory of Environment and Genes Related to Diseases, Ministry of Education, Xi'an, Shaanxi 710061, P.R. China

^3^ Key Laboratory for Tumor Precision Medicine of Shaanxi Province, The First Affiliated Hospital of Xi'an Jiaotong University, Xi’an, Shaanxi 710061, P.R. China

^4^ These authors contributed equally

* Correspondence and requests for materials should be addressed to B.M. (email: bohanma@xjtu.edu.cn) and L.L. (e-mail: [lilydr@163.com)](mailto:lilydr@163.com)).

**This file includes:**

Supplementary Figures S1-S6

Supplementary Table S1-S7


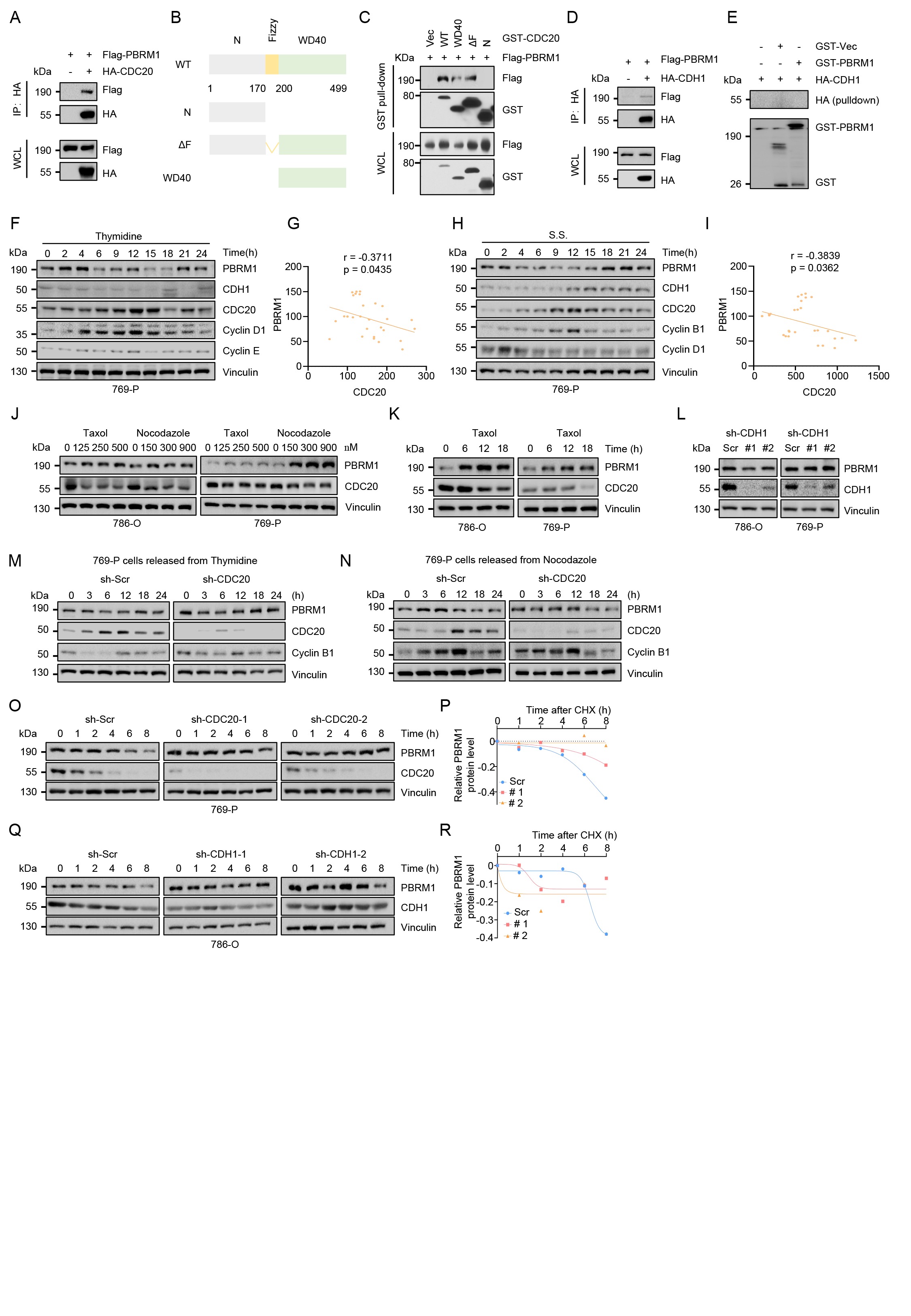


**Supplementary Figure 1. PBRM1 fluctuates during cell cycle progression.** (A) IB analysis of WCL and anti-HA immunoprecipitates derived from 293T cells transfected with HA-CDC20 and Flag-PBRM1. (B) Schematic representation of truncated constructs of CDC20 for mapping the interaction domain with PBRM1. (C) IB analysis of WCL and GST-pull-down products derived from 293T cells transfected with Flag-PBRM1 and indicated constructs of GST-CDC20. Vec, vector. (D) IB analysis of WCL and anti-HA immunoprecipitates derived from 293T cells transfected with HA-CDH1 and Flag-PBRM1. (E) IB analysis of WCL and GST-pull-down products derived from 293T cells transfected with HA-CDH1 and indicated constructs of GST-PBRM1. Vec, vector. (F) IB analysis of WCL derived from 769-P cells synchronized by thymidine block, following by releasing back into the cell cycle for the indicated times. (G) Quantification of CDC20 and PBRM1 blot intensity using the ImageJ software, according to Figure S1F. Data are statistics of three independent experiments. CDC20 and PBRM1 bands were normalized to vinculin. (H) IB analysis of WCL derived from 769-P cells synchronized by serum starvation (S.S.), following by releasing back into the cell cycle for the indicated times. (I) Quantification of CDC20 and PBRM1 blot intensity using the ImageJ software, according to Figure S1H. Data are statistics of three independent experiments. CDC20 and PBRM1 bands were normalized to vinculin. (J) Indicated protein level of 786-O and 769-P cells treated with indicated concentration of taxol and nocodazole for 24 h were analyzed by immunoblotting. (K) IB analysis of WCL derived from 786-O and 769-P cells treated with taxol for the indicated times. (L) Cell lysates derived from 786-O and 786-P cells stably expressing shCDH1 or Scr. Scr, Scramble. (M) IB analysis of WCL derived from WT or CDC20 knockdown 769-P cells synchronized by thymidine block, following by releasing back into the cell cycle for the indicated times. Scr, Scramble. (N) IB analysis of WCL derived from WT or CDC20 knockdown 769-P cells synchronized by nocodazole block, following by releasing back into the cell cycle for the indicated times. Scr, Scramble. (O-P) IB analysis of cell lysates of wild-type (WT or CDC20 knockdown 769-P cells treated with cycloheximide (CHX, 20 μg/ml) at indicated time points. (Q-R) IB analysis of cell lysates of WT or CDH1 knockdown 786-O cells treated with cycloheximide (CHX, 20 μg/ml) at indicated time points.


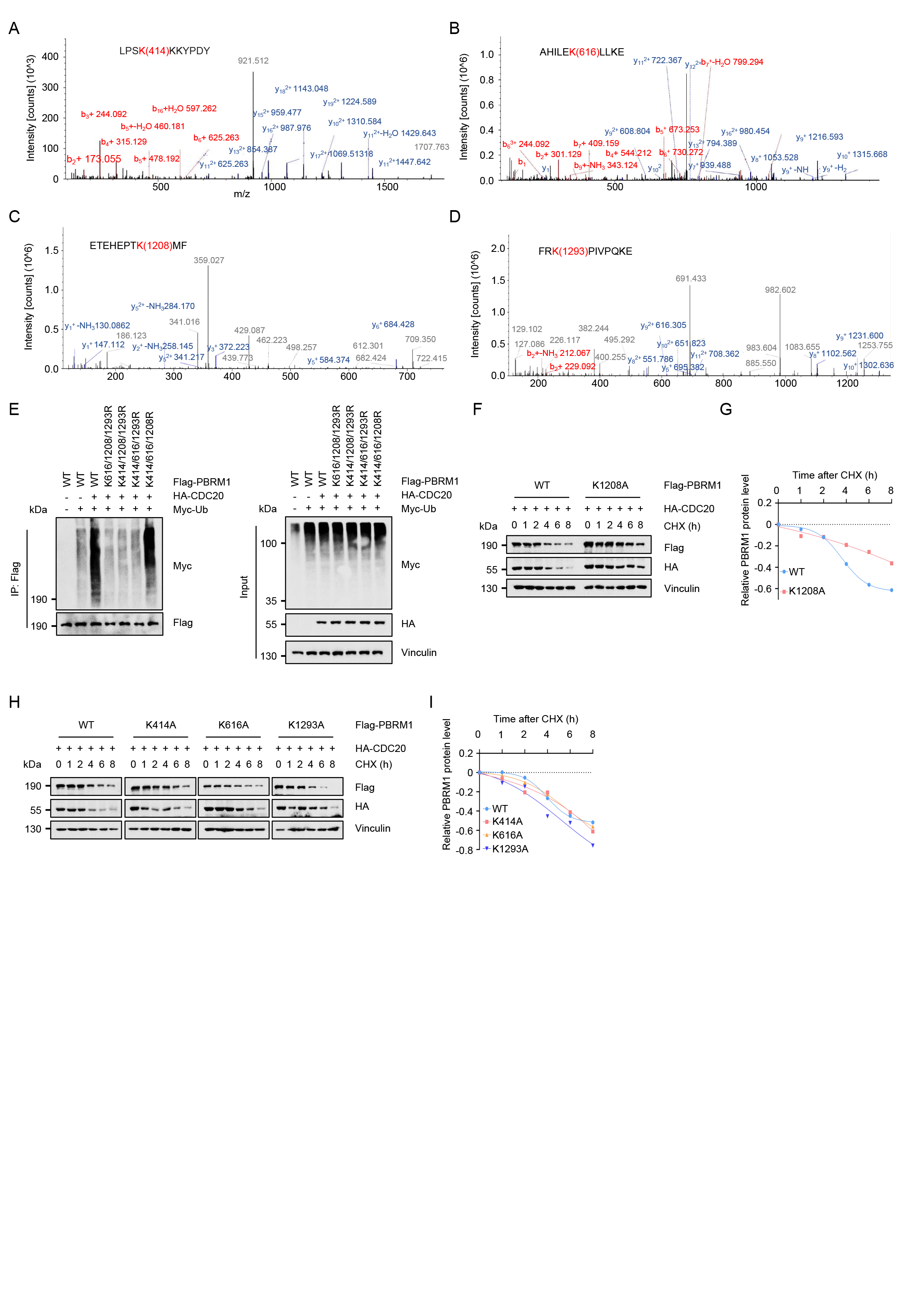


**Supplementary Figure 2. APC-CDC20 E3-ligase complex catalyzes the polyubiquitination of PBRM1.** (A-D) Tandem mass spectrometry spectrum analysis of anti-Flag immunoprecipitates derived from 293T cells transfected with HA-CDC20 and Flag-PBRM1. (E) Immunoblotting analysis of WCL and anti-Flag immunoprecipitates derived from 293T cells transfected with indicated constructs. (F-G) IB analysis of WCL derived from 293T cells transfected with HA-CDC20, Flag-PBRM1 wild-type (WT) and K1208A mutant plasmids. 24 h post transfection, cells were treated with CHX (20 μg/ml) at indicated time points. (H-I) IB analysis of WCL derived from 293T cells transfected with HA-CDC20, Flag-PBRM1 wild-type (WT), K414A, K616A and K1293A mutant plasmids. 24 h post transfection, cells were treated with CHX (20 μg/ml) at indicated time points.


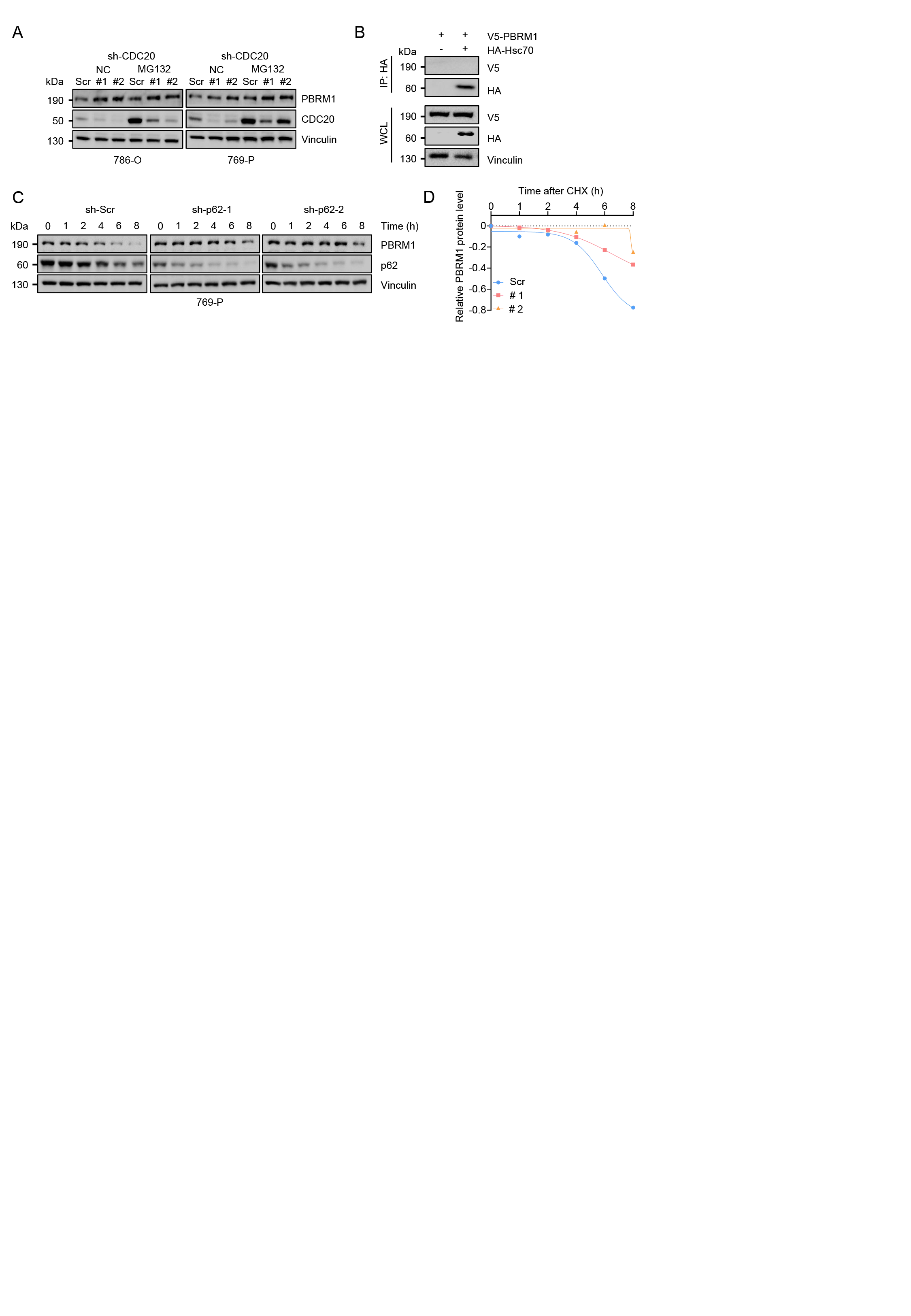


**Supplementary Figure 3. p62 is required for the regulation of PBRM1 by CDC20.** (A) IB analysis of WCL lysates derived from wild-type (WT) or CDC20 knockdown 786-O and 769-P cells treated with MG132 (10 μM) for 12 h before harvesting. (B) IB analysis of WCL and anti-HA immunoprecipitates derived from 293T cells transfected with HA-HSC70 and V5-PBRM1. (C-D) Immunoblotting analysis(C) and quantification(D) of cell lysates of WT and p62 knockdown 769-P cells treated with cycloheximide (CHX, 20 μg/ml) at indicated time points.


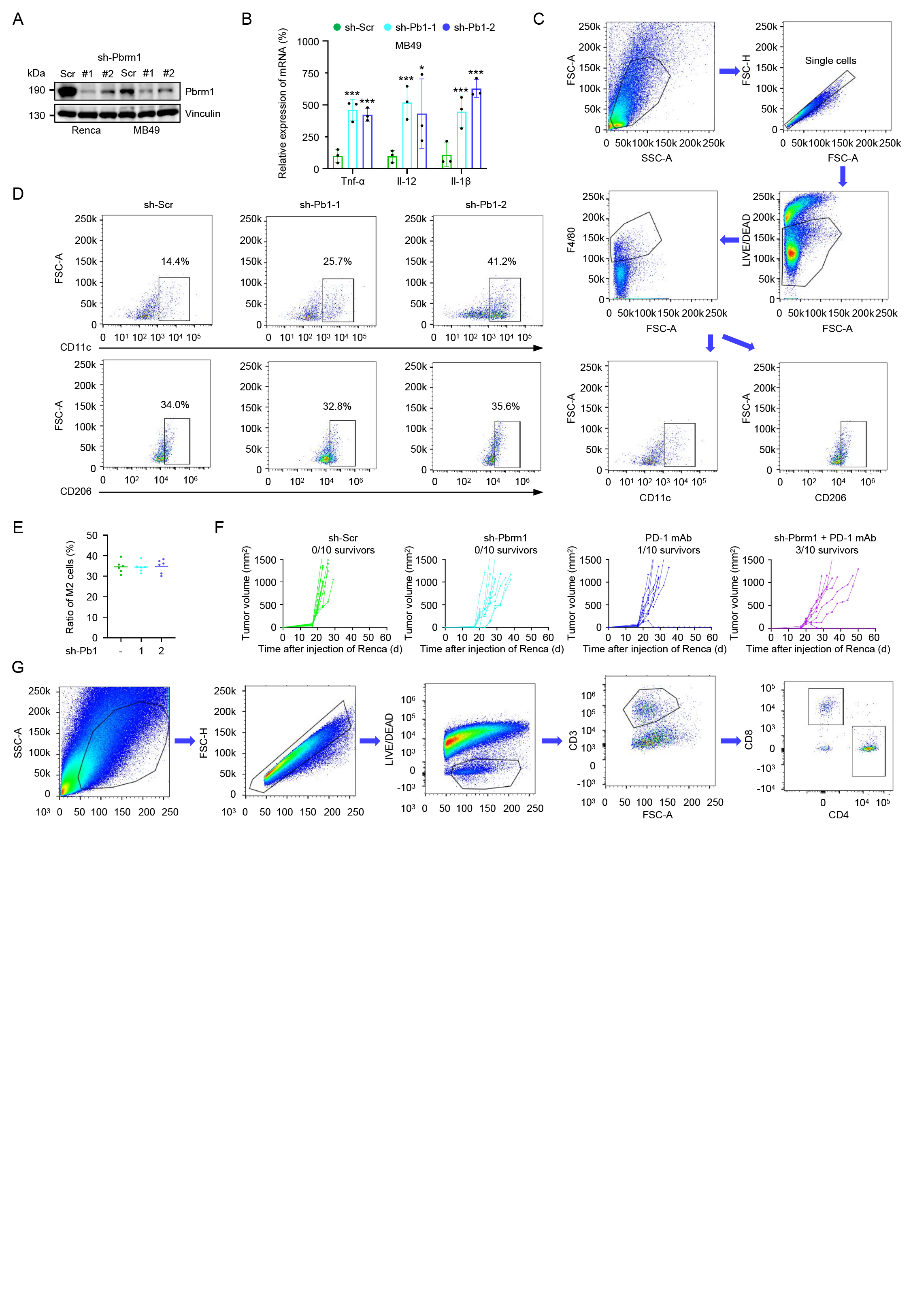


**Supplementary Figure 4. PBRM1 promotes M2 repolarisation to M1.** (A) Validation of PBRM1 knockdown in renca and MB49 cells by immunoblot (IB). (B) TNFα, IL-12 and IL-1β mRNA expression of RAW264.7 after treatment with conditioned medium derived from wild-type (WT) or Pbrm1 knockdown MB49 cells was determined by qTR-PCR. n=3. (C) Flow cytometry gating strategy to identify macrophages. Relevant for Figure S4D and S6H. (D) Representative dot plots M1 and M2 macrophage in renca syngeneic tumors. n=6. (E) Proportions of M2 macrophage infiltrated in wild-type (WT) or Pbrm1 knockdown Renca syngeneic tumors. n = 6. (F) Volumes of wild-type (WT) and Pbrm1 knockdown renca xenograft tumors treated with control or anti-PD-1 mAb (2 mg per mice, three times). n = 10. (G) Flow cytometry gating strategy to identify T cells. Relevant for Figure 4L and 5O.


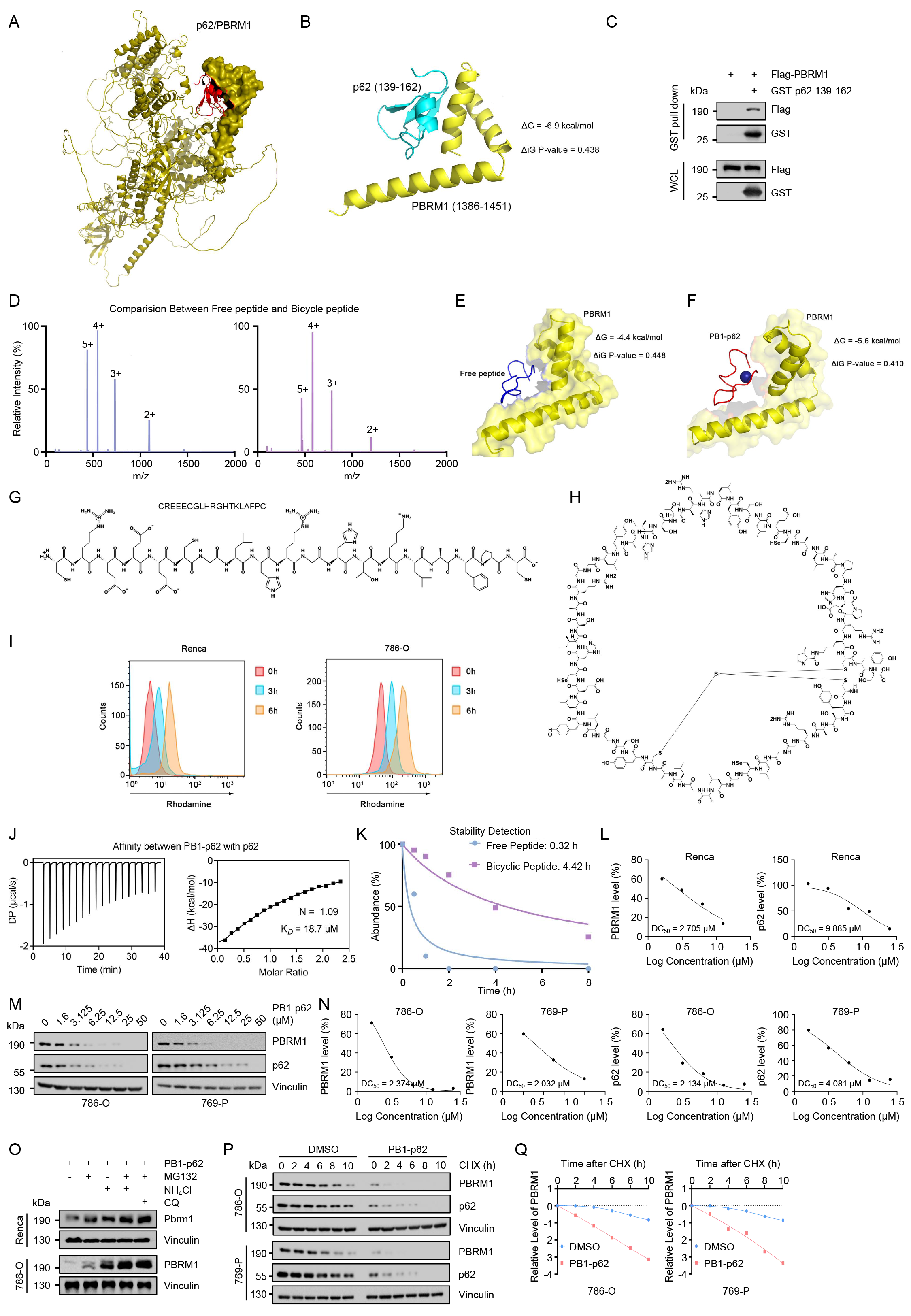


**Supplementary Figure 5. The PB-p62 drug selectively degrades PBRM1.**

1. Prediction of the p62 and PBRM1 complex structure by Zdock. (B) Interface analysis of p62 and PBRM1 conducted using PDBePISA. (C) IB analysis of WCL and GST-pull-down products derived from 293T cells transfected with Flag-PBRM1 and indicated constructs of GST-p62. (D) Mass spectrometry analysis of the designed linear peptide of PBRM1 and the bicyclic peptide after completion of the reaction. (E-F) ZDOCK analysis of interaction between free peptide and PB1-p62 with PBRM1 in renca and 786-O cells, respectively. (G-H) The chemical structure of the free peptide and PB1-p62. (I) Flow cytometry analysis of the uptake ability of PB1-p62 in Renca and 786-O cells. (J) Interaction between the PB1-p62 polypeptide and p62 protein detected by isothermal titration calorimetry (ITC). (K) Serum stability analysis for linear peptides and bicyclic peptide drugs targeting PBRM1. (L) The half-maximal degradation concentration (DC_50_) values of the PB1-p62 drug for Pbrm1 and p62 in renca cells. (M) IB analysis of PBRM1 and p62 in 786-O and 769-P cells after 24 h of treatment with the PB-p62 drug. (N) The half-maximal degradation concentration (DC_50_) values of the PB1-p62 drug for PBRM1 and p62 in 786-O and 769-P cells. (O) IB analysis of WCL lysates derived from renca and 786-O cells treated with NH_4_Cl (20 mM, 24 hours), chloroquine (CQ, 50 μM, 24 hours) or MG132 (10 μM, 12 hours) before harvesting. (P-Q) IB analysis of cell lysates of 786-O and 769-P cells treated with PB-p62 drug (5 μM) for 24 h before treated with cycloheximide (CHX, 20 μg/ml) at indicated time points.


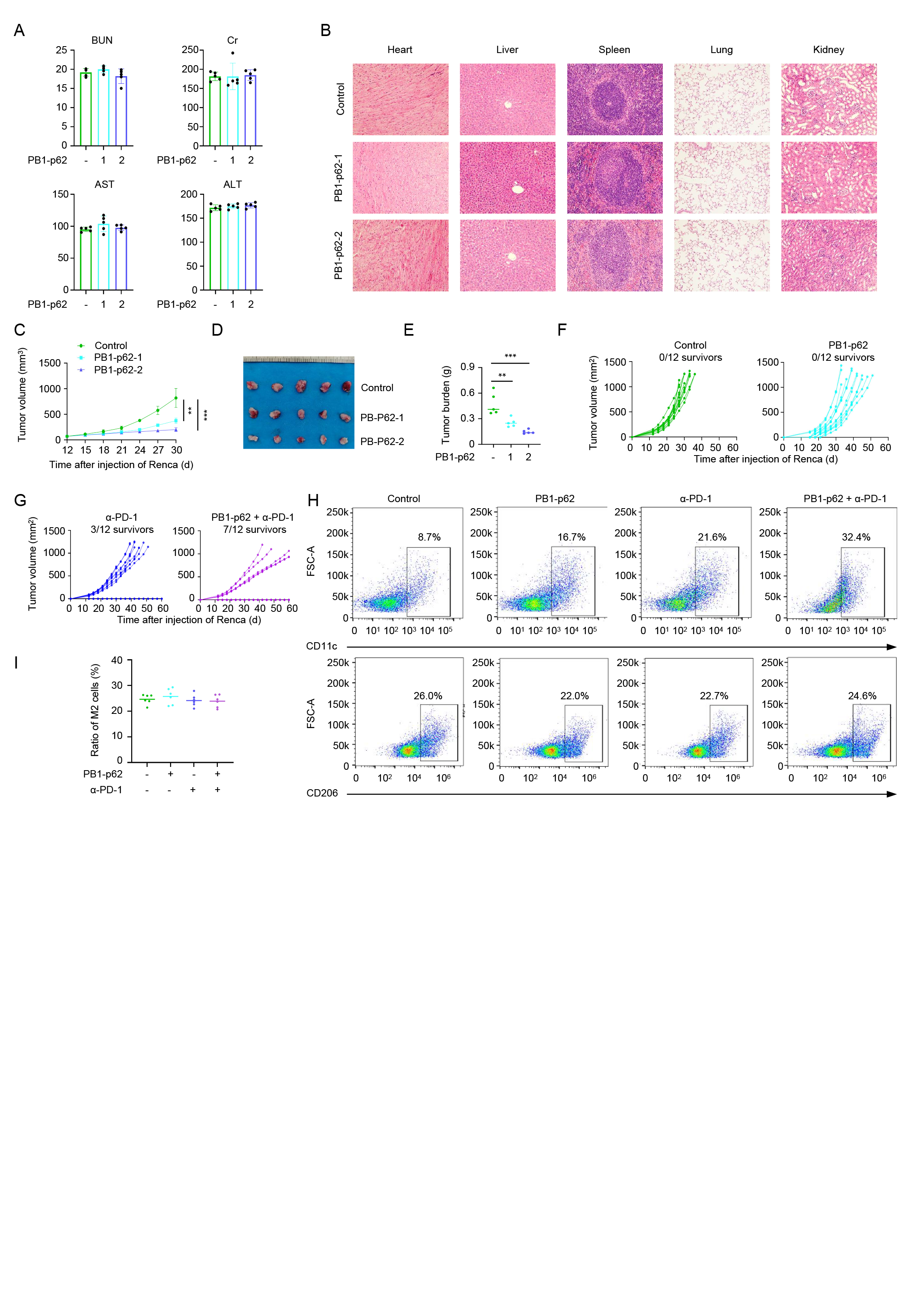


**Supplementary Figure 6. The PB-p62 drug augments immunotherapeutic effects.** (A) Biosafety evaluation of PB1-p62 after a 21-day treatment period. Serum biochemical analysis of BUN (blood urea nitrogen), Cr (serum creatinine), ALT (alanine transaminase) and AST (aspartate transaminase) levels to assess potential organ toxicity. PB1-p62-1: 1 mg/kg for seven times; PB1-p62-2: 2 mg/kg for seven times. (B) Representative H&E staining photographs of heart, liver, spleen, lung and kidney sections from mice after 21 days of treatment with the Control (PBS) or PB1-p62. PB1-p62-1: 1 mg/kg for seven times; PB1-p62-2: 2 mg/kg for seven times. (C) Volumes of renca xenograft tumors treated with PB1-p62. n=5. PB1-p62-1: 1mg/kg for seven times; PB1-p62-2: 2mg/kg for seven times. (D) Tumor size of renca xenograft tumors treated with PB1-p62. n=5. PB1-p62-1: 1 mg/kg for seven times; PB1-p62-2: 2 mg/kg for seven times. (E) Tumor burden of renca xenograft tumors treated with PB1-p62. n=5. PB1-p62-1: 1mg/kg for seven times; PB1-p62-1: 2mg/kg for seven times. (F-G) Volumes of renca xenograft tumors treated with PB1-p62 (2 mg/kg, seven times) and anti-PD-1 mAb (2 mg per mice, three times) either alone or in combination. n = 12. (H) Representative dot plots M1 and M2 macrophage in renca syngeneic tumors. n=6. (I) Proportions of M2 macrophage infiltrated in wild-type (WT) or Pbrm1 knockdown Renca syngeneic tumors. n = 6. Mice were intraperitoneally injected with PB1-p62.

Supplementary Table S1. List of primers

| **Target** | **Sequence** |
| --- | --- |
| Actb-F | GGCTGTATTCCCCTCCATCG |
| Actb-R | CCAGTTGGTAACAATGCCATGT |
| Tnf-α-F | CCCTCACACTCAGATCATCTTCT |
| Tnf-α-R | GCTACGACGTGGGCTACAG |
| Il-12-F | CTGTGCCTTGGTAGCATCTATG |
| Il-12-R | GCAGAGTCTCGCCATTATGATTC |
| Il-1β-F | GCAACTGTTCCTGAACTCAACT |
| Il-1β-R | ATCTTTTGGGGTCCGTCAACT |

Supplementary Table S2. Tumor volume inhibitory rates, related to Figure 4I

| **Groups** | **Number**  **(n)** | **Tumor volume**  **(cm^3^)** | **Inhibitory rate**  **(%)** |
| --- | --- | --- | --- |
| CTRL | 5 | 975.96±174.21 | - |
| shPb1 | 5 | 670.35±150.58 | 31.31 |
| α-PD-1 | 5 | 340.63±40.56 | 65.10 |
| α-PD-1+shPb1 | 5 | 114.83±23.68 | 88.23 |
| α-PD-1+CL | 5 | 444.68±138.25 | 54.44 |
| α-PD-1+shPb1+CL | 5 | 357.73±138.20 | 63.35 |

Supplementary Table S3. Tumor weight inhibitory rates, related to Figure 4K

| **Groups** | **Number**  **(n)** | **Tumor weight**  **(g)** | **Inhibitory rate**  **(%)** |
| --- | --- | --- | --- |
| CTRL | 5 | 0.62±0.09 | - |
| shPb1 | 5 | 0.42±0.07 | 31.91 |
| α-PD-1 | 5 | 0.24±0.06 | 60.81 |
| α-PD-1+shPb1 | 5 | 0.12±0.02 | 80.18 |
| α-PD-1+CL | 5 | 0.42±0.10 | 32.78 |
| α-PD-1+shPb1+CL | 5 | 0.39±0.14 | 37.18 |

Supplementary Table S4. Tumor volume inhibitory rates, related to Figure S6C

| **Groups** | **Number**  **(n)** | **Tumor volume**  **(cm^3^)** | **Inhibitory rate**  **(%)** |
| --- | --- | --- | --- |
| Control | 5 | 819.40±184.49 | - |
| PB1-p62-1 | 5 | 375.15±51.67 | 54.22 |
| PB1-p62-2 | 5 | 200.54±36.43 | 75.53 |

Supplementary Table S5. Tumor weight inhibitory rates, related to Figure S6E

| **Groups** | **Number**  **(n)** | **Tumor weight**  **(g)** | **Inhibitory rate**  **(%)** |
| --- | --- | --- | --- |
| Control | 5 | 0.79±0.22 | - |
| PB1-p62-1 | 5 | 0.42±0.09 | 46.34 |
| PB1-p62-2 | 5 | 0.25±0.04 | 68.97 |

Supplementary Table S6. Tumor volume inhibitory rates, related to Figure 5K

| **Groups** | **Number**  **(n)** | **Tumor volume**  **(cm^3^)** | **Inhibitory rate**  **(%)** |
| --- | --- | --- | --- |
| Control | 6 | 1180.57±540.13 | - |
| PB1-p62 | 6 | 413.58±115.79 | 64.97 |
| α-PD-1 | 6 | 448.18±127.85 | 62.04 |
| α-PD-1+PB1-p62 | 6 | 158.08±66.21 | 86.61 |

Supplementary Table S7. Tumor weight inhibitory rates, related to Figure 5M

| **Groups** | **Number**  **(n)** | **Tumor weight**  **(g)** | **Inhibitory rate**  **(%)** |
| --- | --- | --- | --- |
| Control | 6 | 0.92±0.31 | - |
| PB1-p62 | 6 | 0.53±0.15 | 42.33 |
| α-PD-1 | 6 | 0.38±0.12 | 58.95 |
| α-PD-1+PB1-p62 | 6 | 0.15±0.06 | 83.44 |
